# Seasonal Fluctuations of the Seagrass Holobiont under Contrasting Environmental Conditions

**DOI:** 10.1111/1758-2229.70239

**Published:** 2026-01-23

**Authors:** Tamar Jamieson, Mohsen Chitsaz, Angélique Gobet, Michelle Waycott, Sophie C. Leterme

**Affiliations:** ^1^ College of Science and Engineering, Flinders University Bedford Park South Australia Australia; ^2^ ARC Training Centre for Biofilm Research and Innovation, Flinders University Bedford Park South Australia Australia; ^3^ MARBEC, Univ Montpellier, CNRS, Ifremer, IRD Sète France; ^4^ School of Biological Sciences, Faculty of Science, the University of Adelaide Adelaide South Australia Australia; ^5^ State Herbarium of South Australia, Botanic Gardens and State Herbarium Adelaide South Australia Australia

## Abstract

Microbial communities are widely recognised as indicative of ecosystem health. Changes in the microbial community composition of seagrasses and their environment could act as an important bio‐indicator for stress factors affecting the submerged aquatic plants that make up the *Ruppia* community in the Coorong. Here, we explored prokaryotes associated with surface biofilms of the leaves and roots of the seagrasses to determine the microbiota composition of the *Ruppia* community, and their link to the surrounding sediment and water. *Ruppia* was recorded growing at 55% of the sites surveyed, and all collected samples showed a high diversity of prokaryotes. Turbidity was the main driver of the fluctuations in microbiota composition of the *Ruppia* community. Water and sediment microbial communities were correlated with the presence/absence of the seagrasses. Seagrass health indicators were assessed, allowing for a clear distinction between the various states of the Ruppia community identified in this study. This study provides key baseline insights into the composition and possible functions of these biofilm microbiota, as well as identifying potential health bio‐indicators for the *Ruppia* community. Furthermore, it identifies specific beneficial bacteria that could be selected to enhance seagrass restoration efforts as well as inhibit detrimental algal blooms in the Coorong.

## Graphical abstract

(A) The inharmonious state of the Coorong and the different sample types collect with throughout this study. (B, C) Biofilm, including the presence of diatoms and bacteria, attached on the leaves of Ruppia.

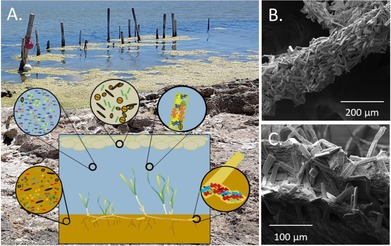

## 1 Introduction

The Coorong holds significant cultural, environmental, and economic value at local, national, and global levels, but has experienced a long‐term decline in its ecological condition due to long‐term reduction in flows from the River Murray (Krull et al. 2009). Seagrasses such as *Ruppia tuberosa* and *Althenia cylindrocarpa*, key components of the Coorong *Ruppia* community, are critical to the aquatic system as ecosystem engineers that are primary producers as well as a food source and shelter for the benthic fauna (Tarquinio et al. 2019). The Coorong naturally shows a strong salinity gradient, fluctuating from fresh water in the northern lagoon to hypersaline in the southern lagoon. The Millennium Drought, particularly during the period 2001–2010, caused severe reductions in freshwater inflows from the River Murray to the Coorong and resulted in a decline in its ecological condition (Mosley and Hipsey 2019). This was particularly evident in the South Lagoon of the Coorong, which experienced extreme increases in salinity (Leterme et al. 2015), a widespread loss of aquatic vegetation and declines in the diversity and abundance of fish, waterbirds, and macroinvertebrates (Brookes et al. 2018). An almost complete decline of the *Ruppia* community occurred in the Coorong during the Millennium Drought and included the loss of seed banks (Waycott and Lewis 2022). The recovery of the *Ruppia* community has been gradual, with the number of sites occupied by plants increasing over the past decade (Lewis et al. 2022). Recovery of the *Ruppia* community has been impeded most strongly in the South Lagoon because of the hypereutrophic state of the southern Coorong (Waycott et al. 2022). The South Lagoon is dominated by algal blooms, including mat‐forming filamentous algae that physically disrupt the seagrasses' ability to flower and set seed (Mosley and Hipsey 2019; Lewis et al. 2022). The microbiota associated with the algal blooms and hypereutrophic conditions in the Coorong are very poorly understood due to the unique set of contextual characteristics of this environment.

Microbial communities are typically indicative of ecosystem health, and dysbiosis has been observed due to large‐scale nutrient shifts in other systems (Hemraj et al. 2017b, 2017a; Cotner and Biddanda 2002; Crossetti et al. 2008; Kiersztyn et al. 2019). Understanding how contextual characteristics affect the stability of microbial community composition is crucial for improving ecosystem management and maintenance. Seagrasses and their associated microbiota altogether can also be referred to as a “holobiont” whereby the functional unit responds to ecological changes as a whole (Simon et al. 2019; Ugarelli et al. 2017; Szitenberg et al. 2022; Egan et al. 2013). Seagrass microbiota are important for the fitness, growth, survival of plants and resistance to stressors (Tarquinio et al. 2019; Seymour et al. 2018; Vandenkoornhuyse et al. 2015), and sections of the seagrass plant have distinct bacterial communities commonly associated with them. Seagrass microbiota provision to the host includes nutrient supply (e.g., nitrogen fixation associated with leaves and roots) and detoxification from harmful compounds (e.g., hydrogen sulfides around roots). Indeed, nitrogen‐fixing bacteria are estimated to provide up to 50% of the nitrogen required by seagrasses (O'Donohue et al. 1991), while sulfate‐oxidising bacteria alleviate the roots and rhizomes from the toxic effects of hydrogen sulfide (Martin et al. 2019). It has been hypothesized that nitrogen‐fixing prokaryotes found both in the phyllosphere (Agawin et al. 2016) and the rhizosphere of seagrasses can provide 30%–100% of their nitrogen requirement (Welsh et al. 2000; Cole and McGlathery 2012; Sun et al. 2015). The microbial community composition of the seagrasses and of its environment could be a useful bio‐indicator for environmental changes that may cause stress to plants in the *Ruppia* Community.

The presence of filamentous algae in the Coorong is an important contributor to sediment nutrient loads at certain times of the year and has a negative impact on the *Ruppia* community (Waycott et al. 2022). High NH_4_ levels in sediment pore water and high flux rates to the water column from the South Lagoon, which provides the “fuel” for filamentous algal growth, have been reported throughout 2020–2021 (Huang et al. 2022). Excessive filamentous algal growth in the Coorong, which begins in late spring and continues through the summer, adversely affects Ruppia growth and seed production throughout the system. These impacts are particularly noticeable in the southern Coorong, leading to further negative consequences for the entire Coorong ecosystem (Waycott et al. 2022; Collier et al. 2017; Brookes et al. 2021). These impacts are hampering the long‐term recovery of the Coorong ecosystem following the large‐scale losses of the *Ruppia* Community in the Coorong during the Millennium Drought (Collier et al. 2017; Rogers and Paton 2009) despite translocation efforts that led to recruitment of Ruppia across areas where seeds were deployed in sediments (Waycott et al. 2022; Collier et al. 2017; Paton, Bailey, et al. 2018; Paton, Paton, and Bailey 2018). The current state of the previously robust *Ruppia* community, was described in the findings of the Goyder expert panel review (Brookes et al. 2018) as being in a vulnerable condition requiring significant effort to restore resilience and associated ecological functions.

Here, we identified the prokaryotic community associated with surface biofilms of the leaves and the roots of plants in the *Ruppia* community, as well as of the surrounding sediment, water and algal mats, to determine the impact contextual conditions in the Coorong have on this microbial community. We also assessed the applicability of the seagrass health indicators determined by Martin et al. (2020) to determine if they could reflect the different health states of the *Ruppia* community observed in the Coorong.

## 2 Materials and Methods

### 2.1 Sampling Sites

**FIGURE 1:**
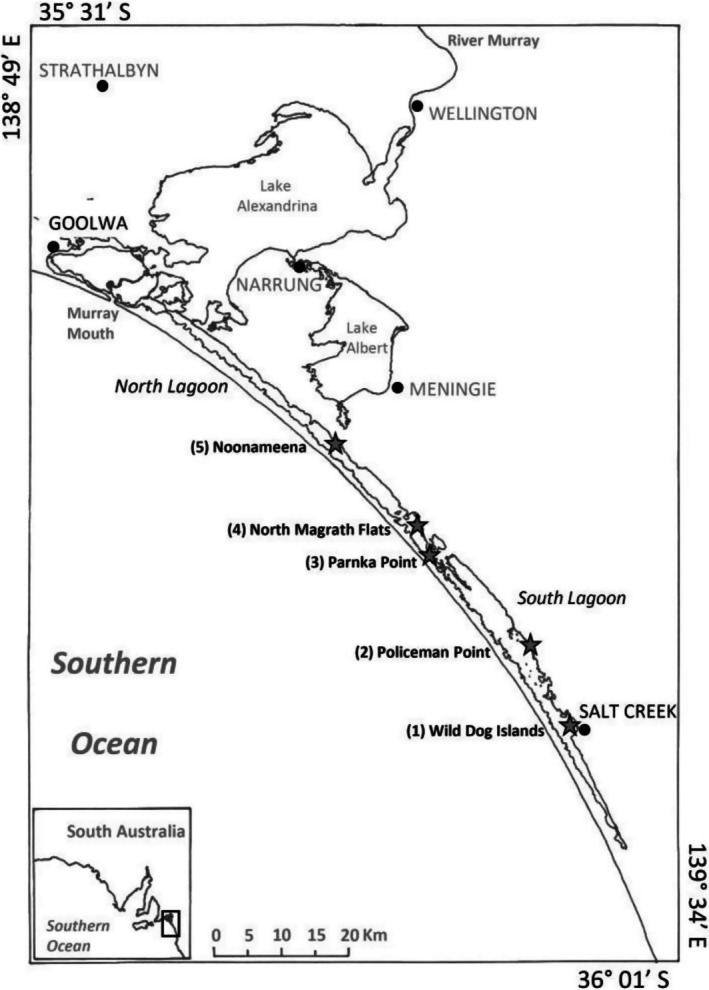
Map of the sampling sites—Coorong South Lagoon: Site 1, Wild Dog Islands; Site 2, Policeman Point. Central Coorong: Site 3, Parnka Point; Site 4, North Magrath Flats. Coorong North Lagoon: Site 5, Noonameena.

Five sites were chosen along the Coorong, from Wild Dog Islands in the South Lagoon to Noonameena in the North Lagoon (Figure 1), as the focus of this study. Sampling was conducted seasonally over a year: Spring (27 October 2020), Summer (16 December 2020), Autumn (9 March 2021), and Winter (15 June 2021). Physico‐chemical parameters including temperature, salinity, dissolved oxygen, turbidity, and dissolved nutrients (NO_x_, NH_4_, PO_4_) were monitored, in triplicates, at each site (Figure S2). Analyses of all dissolved nutrient concentrations were measured simultaneously and carried out following published methods (Leterme et al. 2015), using a SKALAR SFA nutrient analyser.

### 2.2 Water and Sediment Microbiota

Triplicate 2 L water samples were collected for DNA extraction and passed through a 20 μm sieve before being filtered through 0.22 μm Millipore membrane filters (MF‐Millipore Membrane Filters HAWP04700). Filters were placed in sterile and UV‐treated petri dishes tightly closed using Parafilm and stored at −20°C until DNA extraction.

Triplicate samples of 2 mL of surface sediment were collected along the waterline for DNA extraction using a modified 5 mL syringe stored at −20°C until DNA extraction.

### 2.3 The Ruppia Community

Within this study, the *Ruppia* community is considered to be a mix of *Ruppia tuberosa, Althenia cylindrocarpa* and another unresolved species of *Ruppia* (Lewis et al. 2022; Waycott et al. 2022, 2020; Brookes et al. 2021). At each sampling site, three 45 mm diameter cores were taken randomly within a patch of *Ruppia*, to a depth of 10 cm. As plants of the *Ruppia* community were not present at all sampling sites, samples could not always be collected in triplicate. In the laboratory, the *Ruppia* community samples were dissected to separate the root systems from the leaves. When filamentous algae were present amongst the *Ruppia* community, either by forming filamentous aggregates attached to the seagrass plants (PAA) or by forming floating mats in which seagrass plants were trapped, PAA and mats were also sampled.

All collected material was processed using the modified protocol of Ugarelli et al. (Ugarelli et al. 2018). The supernatant was sequentially filtered onto 0.22 μm Millipore membrane filters (MF‐Millipore Membrane Filters HAWP04700). Filters were placed in petri dishes, enclosed using Parafilm and stored at −20°C until DNA extraction.

### 2.4 DNA Extraction, Amplicon Formation, Sequencing, and Bioinformatics

DNA was extracted from the filters using the DNeasy PowerWater Kits (Qiagen, CA, USA). Whereas DNA from the sediment samples (0.25 g) was extracted using the DNeasy PowerSoil Kit (Qiagen, CA, USA). Amplicons were prepared for sequencing by PCR using the primer pair 515F/806R (Ugarelli et al. 2018) and each individual extracted DNA as a template amplifying the prokaryotic V4 region of the 16S rRNA gene. The amplicons were sequenced using the Illumina MiSeq System (2 × 250 bp paired‐end sequencing) at the Australian Genome Research Facility (AGRF, Melbourne, Australia). Sequencing analysis was inferred using the DADA2 package (version 1.22) (Callahan et al. 2016) in R studio v.4.6.1 (R v.4.1.3) using amplicon sequence variants (ASVs) which created groups based on sequence similarities. Taxonomy was inferred (98% sequence similarity) for the 16S rRNA gene sequences using the SILVA database release 138.1 (Quast et al. 2012).

### 2.5 Statistical Analysis

Statistical analysis was undertaken using PRIMER v.7 software +PERMANOVA add on (Clarke and Gorley 2006; Anderson et al. 2008), unless otherwise specified. All samples were collected in triplicates throughout the study.

Species diversity, Pielou's evenness, species richness, and community diversity index were calculated (Figure S1). Dissimilarity between sample types, as well as *Ruppia* conditions was assessed using the SIMPER method (Anderson et al. 2008). Microbial community data was transformed using log(x + 1). A Bray–Curtis similarity matrix was created from the transformed microbial community data and used as input for Principal Coordinate Analysis (PCoA). Significant differences between sample types, sampling sites and seasons were tested using PERMutational ANalysis Of VAriance (PERMANOVA) on the transformed data. Canonical Analysis of Principal coordinates (CAP) based on the Bray‐Curtis similarity matrix was used to plot the discrimination between sites.

Environmental data was normalized to the mean. A Principal Component Analysis (PCA) was used to explore the water quality parameters contributing to the differences between sampling sites. Significant differences were tested using PERMANOVA and the BEST procedure was used to find which combination of environmental variables best explains the patterns seen in the microbial community data.

Functional metabolic or relevant ecological functions were inferred using the FAPROTAX v1.2.6 database and script (Louca et al. 2016) against the ASV assigned taxonomy dataset. Differential abundances between functional groups were compared using the DESeq2 package (version 1.38.3) (Love et al. 2014) for R Software (version 4.2.3, Core Team 2021). Heat maps were created using ComplexHeatmap (Gu et al. 2016) v2.15.4 for R Software (version 4.2.3).

Additional methodological details can be found in the Supporting Information.

## 3 Results and Discussion

### 3.1 Coorong—Physico‐Chemical Properties

**FIGURE 2:**
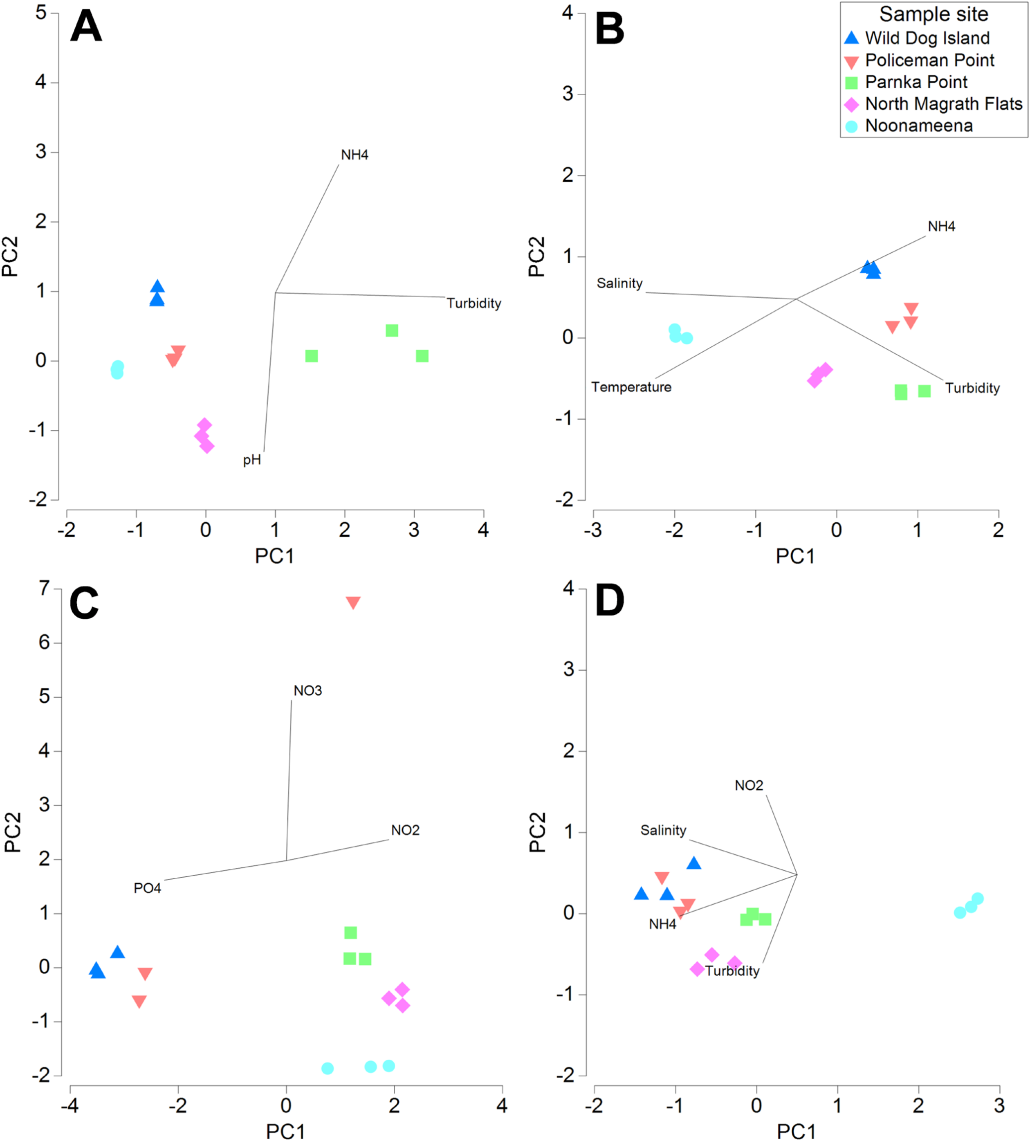
Principal component analysis (PCA) plot for the water quality parameters measured at each of the sampling sites in (A) October 2020 (Trip 1), (B) December 2020 (Trip 2), (C) March 2021 (Trip 3) and (D) June 2021 (Trip 4). Pearson correlation vectors (*r* > 0.8) represent the water quality parameters driving the differences between samples (*n* = 3 per site per trip).

The physico‐chemical parameters showed significant differences between sites and times of sampling (Figure 2). The strongest gradients observed in salinity, dissolved oxygen (DO), and turbidity along the north–south axis of the Coorong were in March 2021. These results are consistent with previous findings (Leterme et al. 2015; Hemraj et al. 2017b, 2017a), with the Coorong ecosystem now recognised as a hyper‐eutrophic environment (Mosley and Hipsey 2019). Alongside these ecological changes, there have been impacts on invertebrates, fish, and waterbirds. Significant differences were observed in the microbial community composition between sample types (water, sediment, leaves, roots, PAA, mats; PERMANOVA *p* < 0.001) and between locations (South lagoon, Central lagoon, North lagoon; PERMANOVA *p* < 0.001). The hyper‐salinity and hyper‐eutrophic conditions of the South Lagoon are likely to have altered the structure of microbial communities and their ecological functions. Dramatic changes to microbial communities have been observed in other systems such as the Great Masurian Lakes (Poland) and the Garças Reservoir (Sãn Paulo, Brazil) experiencing large‐scale nutrient shifts (Hemraj et al. 2017b, 2017a; Cotner and Biddanda 2002; Crossetti et al. 2008; Kiersztyn et al. 2019).

The nutrient levels measured throughout the study characterized a strong deficit in nitrogen, that is, a decrease in the ratio of fixed inorganic nitrogen to phosphorus (N:P) relative to the Redfield ratio (16:1) (Redfield et al. 1963). These results are supported by Huang et al. (2022) who also observed limited nitrate availability within the Coorong and a lack of coupled nitrification–denitrification activity throughout 2020–2021, which results in the removal of nitrogen from the system (in gaseous form). It is often assumed that inland waters have large inputs of organic matter from terrestrial environments with typically high carbon:nitrogen:phosphorus ratios (Hessen et al. 2003), however in the Coorong the main source of nutrients is from adjacent waters. The apparent flushing of nutrients in winter, despite increased nutrient inputs from the Murray‐Darling Basin, is likely due to better mixing with higher water levels (driven by both inflows, tides, and sea level increase in winter), with the net effect of water with lower nutrient concentrations coming into the Coorong, and nutrient‐rich waters leaving the lagoon.

The relationship between water quality and plant condition becomes more evident when the type of plant material at each site is considered. As evident during the sampling trip in March 2020, the highest salinity was noted, yet no plant material was found at any of the five sampled sites.

### 3.2 Composition of the Seagrass Community

**FIGURE 3:**
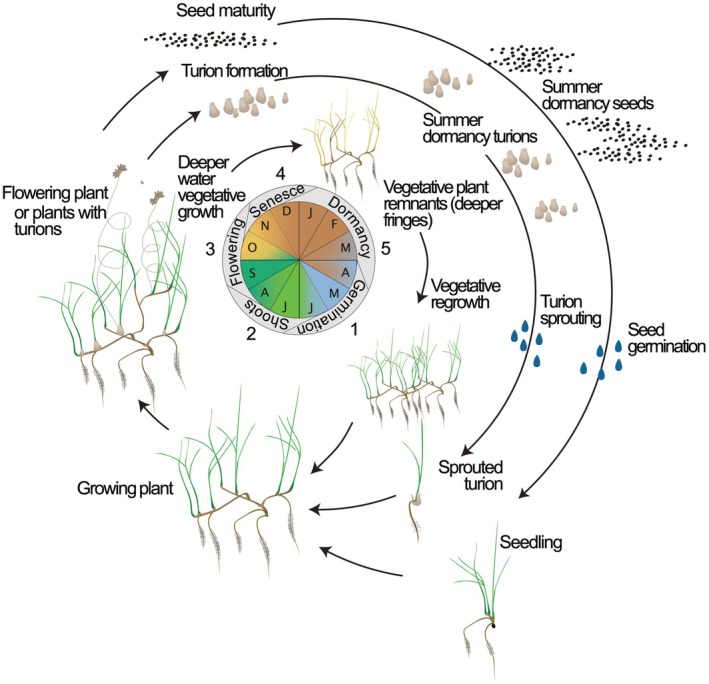
Conceptual diagram of the Ruppia tuberosa life cycle showing annual growth through three possible life cycle pathways: vegetative (whole plant survival); asexual persistence (turions); sexual (seed bank). The diagram also includes the months of the year and each life stage of the *Ruppia* plant they relate to. Source: Asanopoulos and Waycott (2020).

Plants in the *Ruppia* community are known to be present in the Coorong throughout the year (Waycott et al. 2020), with the *Ruppia* community confirmed to include mixed populations of *Ruppia tuberosa* and *Althenia cylindrocarpa* and another unresolved species of *Ruppia* (Lewis et al. 2022; Waycott et al. 2022, 2020; Brookes et al. 2021). The similarity of their simple vegetative form (i.e., thin, long leaves, fine rhizomes, and roots) for these two aquatic plants makes them impossible to differentiate unless when flowering (Lewis et al. 2022; Waycott et al. 2022, 2020). Throughout this paper we refer to this community of macrophytes as the *Ruppia* community. The Spring and Summer sampling events corresponded to the period *Ruppia* or *Althenia* plants were flowering and setting seed (reproductive), while the Autumn sampling event corresponded to aestivation, and the Winter sampling event during the period of vegetative growth. *Ruppia* community leaves and roots were found at all sites in October and December 2020 (Figure 3) and in the Central and North Lagoon in June 2021. However, in March 2021, no leaves were found at any sites except in the North Lagoon.

### 3.3 Microbial Community Structure on Ruppia

**FIGURE 4:**
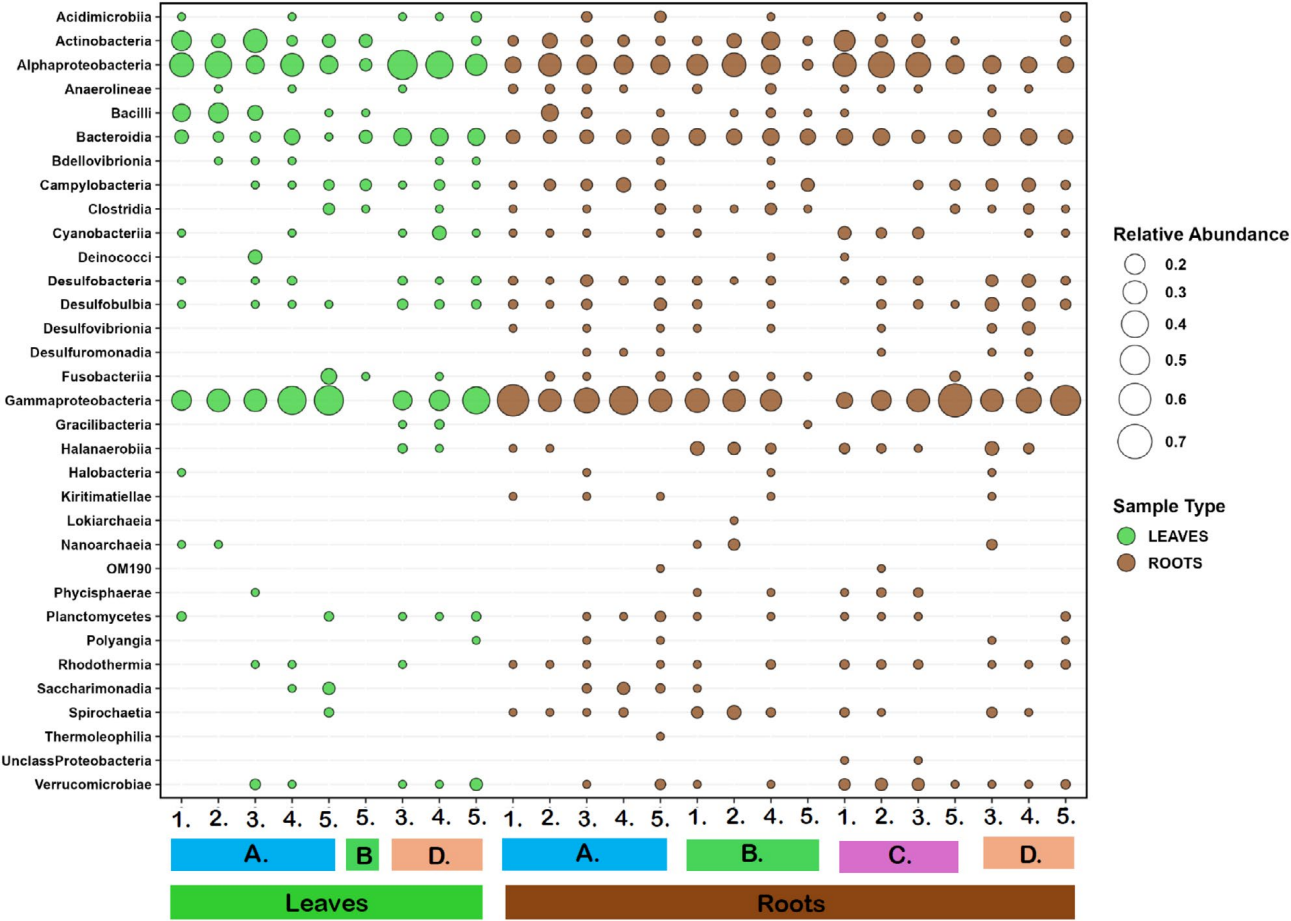
Relative abundance of all the taxa that comprise more than 1% of the archaea and bacterial sequences in the *Ruppia* Community from 16S rRNA gene sequencing at the five sampling sites in October 2020 (A), December 2020 (B), March 2021 (C), and June 2021 (D). Sampling sites—*Coorong South Lagoon*: Site 1, Wild Dog Islands; Site 2, Policeman Point. *Central Coorong*: Site 3, Parnka Point; Site 4, North Magrath Flats. *Coorong North Lagoon*: Site 5, Noonameena. The taxa are classified at the class level according to the Silva database (version 138.1). *n* = 3 per site per trip.

A total of 138 archaea and bacteria classes were identified on the leaves of *Ruppia* community plants, while 137 classes were found on the roots. The dominant bacteria classes were identified as *Gammaproteobacteria* (7.93% (leaves) and 18.24% (roots) relative abundance), *Alphaproteobacteria* (6.82% (leaves) and 9.57% (roots) relative abundance), *Actinobacteria* (1.84% (leaves) and 2.6% (roots) relative abundance) and *Bacteroidia* (2.34% (leaves) and 4.83% (roots) relative abundance), as well as *Bacilli* (1.47% relative abundance) for *Ruppia* leaves only (Figure 4). The classes of *Gammaproteobacteria*, *Alphaproteobacteria*, *Actinobacteria, Verrucomicrobia*, *Planctomycetes and Cyanobacteria* were associated with *Ruppia* leaves (Figure 4), consistent with those identified on other seagrass plants such as *Zostera marina, Zostera noltii*, and *Halophila stipulacea* (Tarquinio et al. 2019).

**FIGURE 5:**
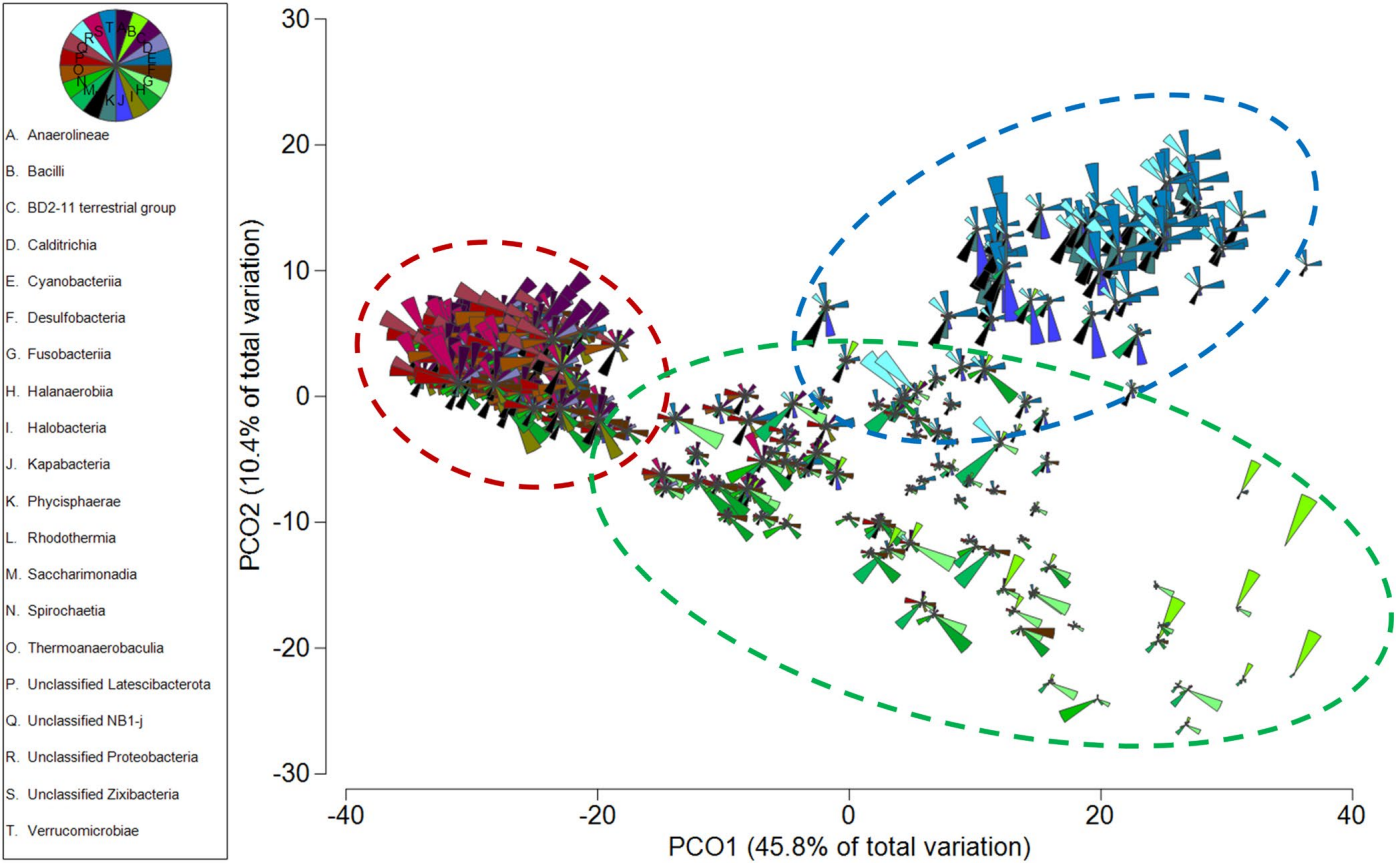
Principal coordinates analysis (PCoA) based on a Bray‐Curtis similarity matrix of the communities of classes of Archaea and Bacteria. Classes explaining most of the dissimilarities (SIMPER analysis) between sample types were used to illustrate the communities of each sample type: water column (blue dotted line), sediment (brown dotted line) and Ruppia community (green dotted line) samples are indicated by the circles on the diagram. Samples were collected across four sampling trips at five sites (*n* = 3 per site per trip).

The results of a SIMPER analysis were used to illustrate the dissimilarities in community composition on the PCoA plot (Figure 5). SIMPER allowed the identification of 20 classes of Archaea and Bacteria that were driving the dissimilarities between sample types as follows: *Anaerolineae, Bacilli, BD2‐11, Calditrichia, Cyanobacteria, Desulfovibrionia, Fusobacteria, Halobacteria (Archaea), Halanaerobiia*, *Kapabacteria, Latescibacteria, Phycisphareae, Rhodothermia, unclassified NB1‐j, unclassified Latescibacteria, unclassified Proteobacteria, unclassified Zixibacteria and Verrucomicrobiae* (Figure S3).

Our findings are consistent with previous studies examining bacteria associated with the root system of a number of different seagrass species with the phyla *Proteobacteria, Actinobacteria, Firmicutes* and *Bacteroides* commonly found (Tarquinio et al. 2019; Liu, Carvalhais, et al. 2017; Bulgarelli et al. 2013; Reinhold‐Hurek et al. 2015; Ferrando and Fernández Scavino 2015; Marques et al. 2015). Seagrasses exhibit strong selectivity in colonising organisms (Liu, Carvalhais, et al. 2017; Bulgarelli et al. 2013; Reinhold‐Hurek et al. 2015), with root exudates like amino acids, organic acids, and sugars influencing the growth of specific microbes (el Zahar Haichar et al. 2014; Somenahally et al. 2011).

**FIGURE 6:**
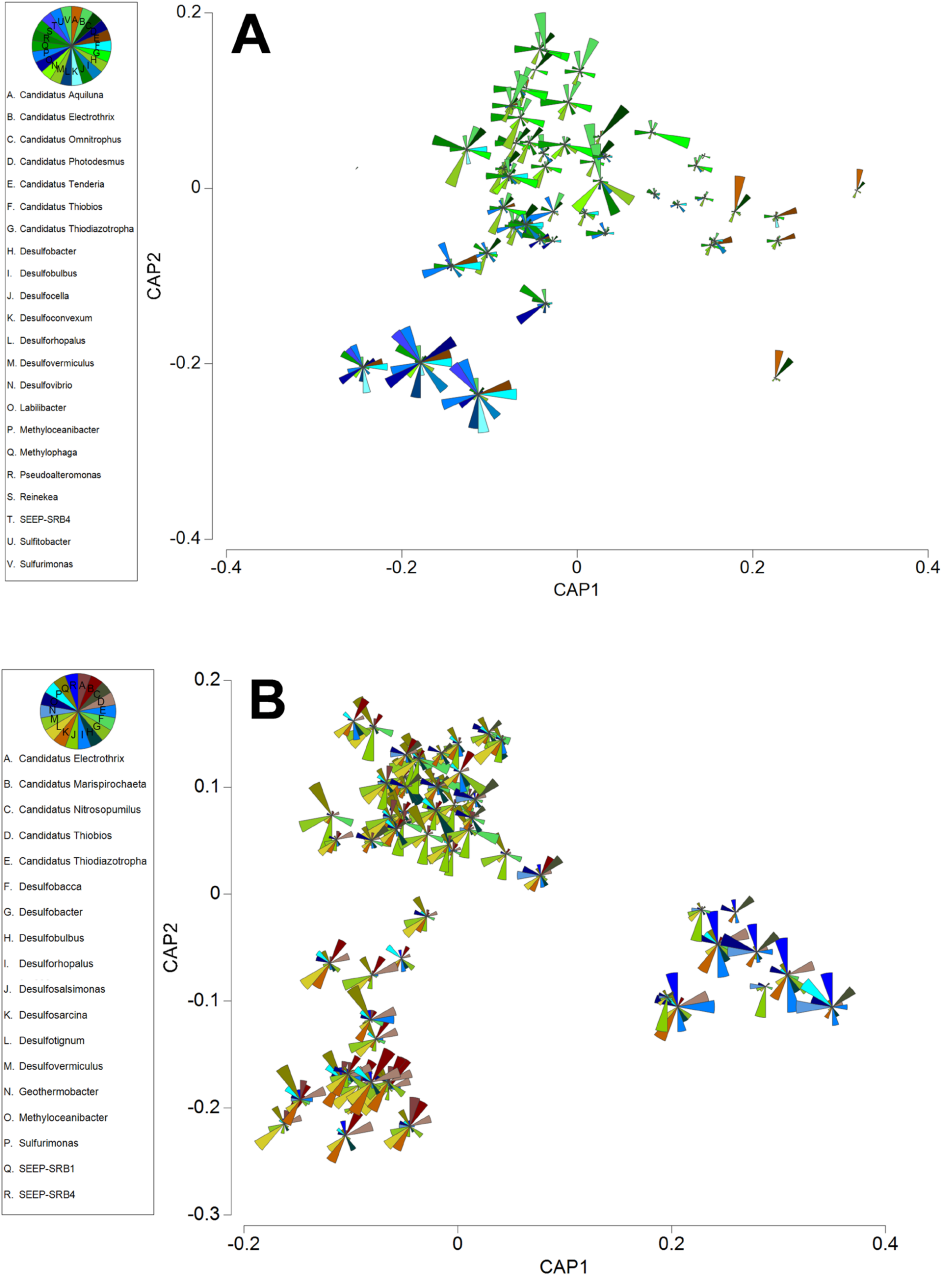
Canonical analysis of principle coordinates (CAP) based on a Bray‐Curtis similarity matrix of the genus of Archaea and Bacteria in the (A) root and (B) sediment microbiota. Genus explaining most of the dissimilarities (SIMPER analysis) between the stages of the life cycle of Ruppia that is, summer dormancy turion (blue shades), vegetative remnants (only roots present before vegetative regrowth) present (brown shades), and growing plant ie. leaves and roots present (green shades). Samples were collected across four sampling trips at five sites (*n* = 3 per site per trip).

The Canonical Analysis of Principal coordinates (CAP) analysis in Figure 6A highlighted a clear spatial organisation of the microbial community, identifying unique associations between bacterial community composition and the different morphological parts of the *Ruppia* plant. Much of the variation in microbial communities could be attributed to the lifecycle stage (Figure 3) of the *Ruppia* plant (i.e., if roots and leaves were present or absent). Numerous genera were either positively or negatively correlated (Pearson > 0.6) with the presence or absence of *Ruppia* roots/leaves. The genera correlating with the presence of vegetative remnants (only roots before vegetative regrowth) were positively correlated, whereas those that were correlated with the absence of *Ruppia* plants were all negatively correlated. *Phaeobacter* was identified as being positively correlated with the presence of vegetative remnants of the *Ruppia* plant. *Phaeobacter* is commonly identified as a growth‐limiting bacterium not only against fish pathogens but also against the formation of harmful algal blooms (Rasmussen et al. 2018; Inaba et al. 2019, 2020). *Phaeobacter* was effective in the inhibition of *Chattonella antiqua* harmful algal blooms in seagrass beds of *Zostera marina* . It is of interest to note that no algal mats and/or algal aggregates were present in any of the sampling sites when vegetative remnants of the *Ruppia* plant were present. Here, the identification of potentially beneficial bacteria associated with the roots of the *Ruppia* community, in a life cycle stage that precede vegetative regrowth, provides avenues for future work on the specific selection of *Phaeobacter* to help prevent algal blooms within the Coorong.

### 3.4 Spatial Patterns of the Bacterial Community Associated With Ruppia

**FIGURE 7:**
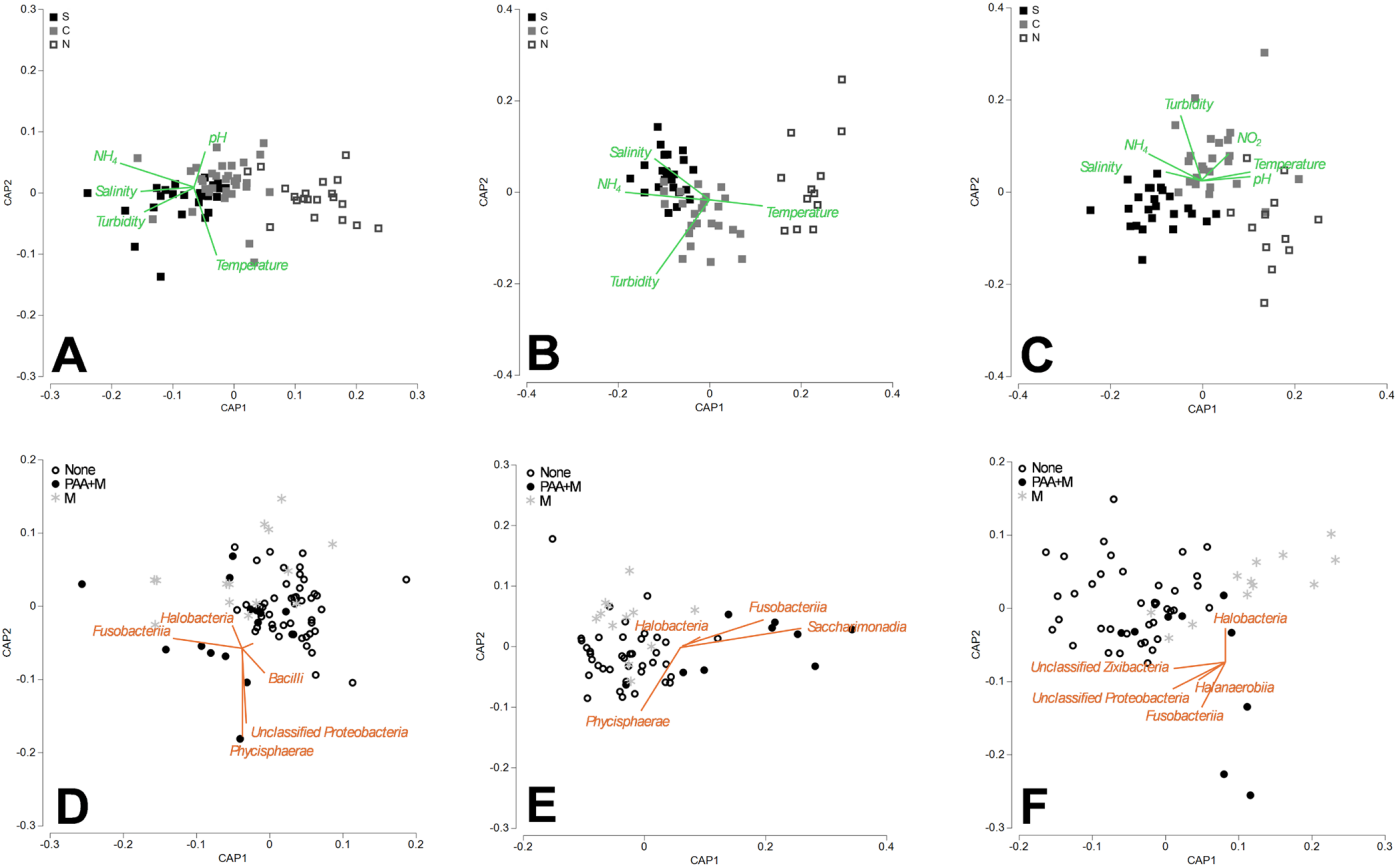
Canonical analysis of principal coordinates (CAP) based on a Bray‐Curtis similarity matrix of the classes of Archaea and Bacteria explaining most of the dissimilarities (SIMPER analysis) detected on *Ruppia* Community (A, D), in water samples (B, E) and in sediment samples (C, F) from 16S rRNA gene sequencing across the Coorong lagoons (A–C) that is, South lagoon (S), Central lagoon (C) and North lagoon (N) over the duration of the study. The impact of the presence of filamentous aggregates attached to the seagrass plants (PAA) or of filamentous algae mats (M) was also investigated (D–F) that is, presence of PAA and mats (PAA+M), presence of mats (M) and absence of PAA and mats (None). (A–C) Pearson correlation vectors (*r* > 0.2) represent the physico‐chemical parameters (green vectors) driving the differences between lagoons. (D–F) Pearson correlation vectors (*r* > 0.3) represent the classes of Archaea and Bacteria (orange vectors) driving the differences between conditions of filamentous algae.

The location of the sampling sites within the Coorong, that is, the environmental conditions present along the north–south axis of the Coorong, was more influential to the observed microbial community fluctuations than seasonal differences (Balzano et al. 2015). Figure 7A–C shows CAP used to discriminate the *Ruppia* microbiota (Figure 7A), water (Figure 7B) and sediment (Figure 7C) between lagoons in the Coorong. Distinctive spatial patterns are evident reflecting the impact of conditions at specific sampling sites on the communities. The CAP1 axis of the CAP analysis distinctively ordinates the communities according to the lagoons as observed for the *Ruppia* microbiota (Figure 7A; CAP 1 correlation *δ*_*1*_^2^ = 0.729, *p* < 0.001), for the water microbiota (Figure 7B; CAP 1 correlation *δ*_*1*_^2^ = 0.887, *p* < 0.001) and for the sediment microbiota (Figure 7C; CAP 1 correlation *δ*_*1*_^2^ = 0.724, *p* < 0.001). The differences observed in the *Ruppia* microbiota between lagoons are explained by the variations in pH, temperature, and turbidity (BEST, Spearman *ρ* = 0.193, *p* < 0.01). In comparison, the differences observed in the microbial communities between lagoons for the water are explained by variations in temperature, salinity, turbidity, and NH₄^+^ (BEST, Spearman *ρ* = 0.350, *p* < 0.01). For the sediment, the differences are explained by variations in temperature and salinity (BEST, Spearman *ρ* = 0.271, *p* < 0.01).

### 3.5 Influence of Contextual Parameters on the Microbial Community Structure and Composition

**FIGURE 8:**
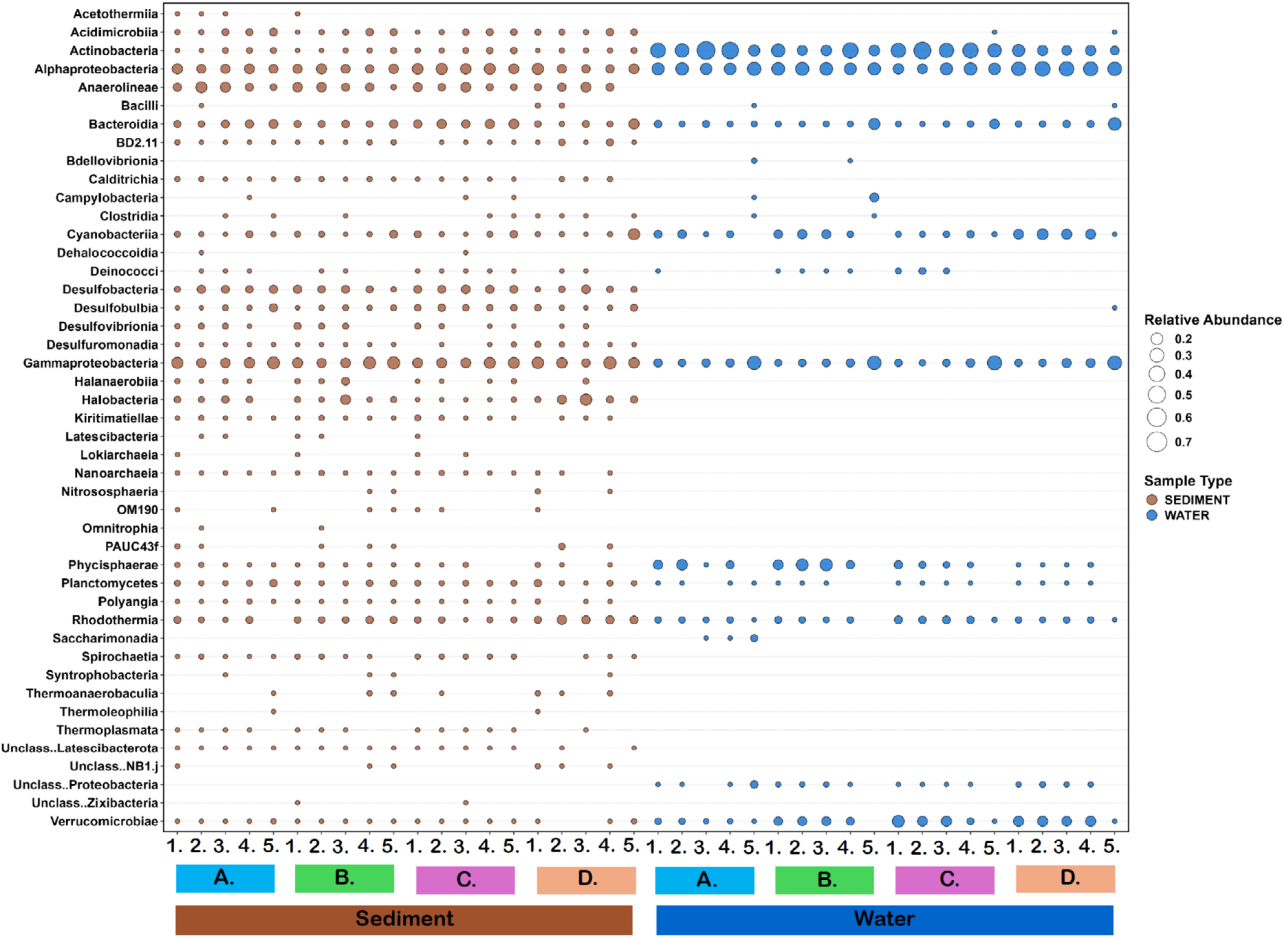
Relative abundance of all the taxa that comprise more than 1% of the archaea and bacterial sequences in the water, and sediment samples, from 16S rRNA gene sequencing at the five sampling sites in October 2020 (A), December 2020 (B), March 2021 (C), and June 2021 (D). Sampling sites—*Coorong South Lagoon*: Site 1, Wild Dog Islands; Site 2, Policeman Point. *Central Coorong*: Site 3, Parnka Point; Site 4, North Magrath Flats. *Coorong North Lagoon*: Site 5, Noonameena. The taxa are classified at the class level according to the Silva database (version 138.1). *n* = 3 per site per trip.

The influence of the surroundings of the *Ruppia* plants was assessed, with 19 archaea and bacteria classes identified in the water, and 42 archaea and bacteria taxa identified in the sediment, throughout the study. The dominant bacterial classes in the water were *Actinobacteria* (5.44% relative abundance), *α‐proteobacteria* (4.89% relative abundance), *γ‐ proteobacteria* (2.31% relative abundance), and *Verrucomicrobiae* (1.58% relative abundance) at all the sites. Within the sediment samples, the dominant bacterial classes were *γ‐proteobacteria* (3.09%), *α‐proteobacteria* (2.49%), *Anaerolineae* (1.62%), *Bacteroidia* (1.4%), and *Desulfobacteria* (1.2%) (Figure 8).

**FIGURE 9:**
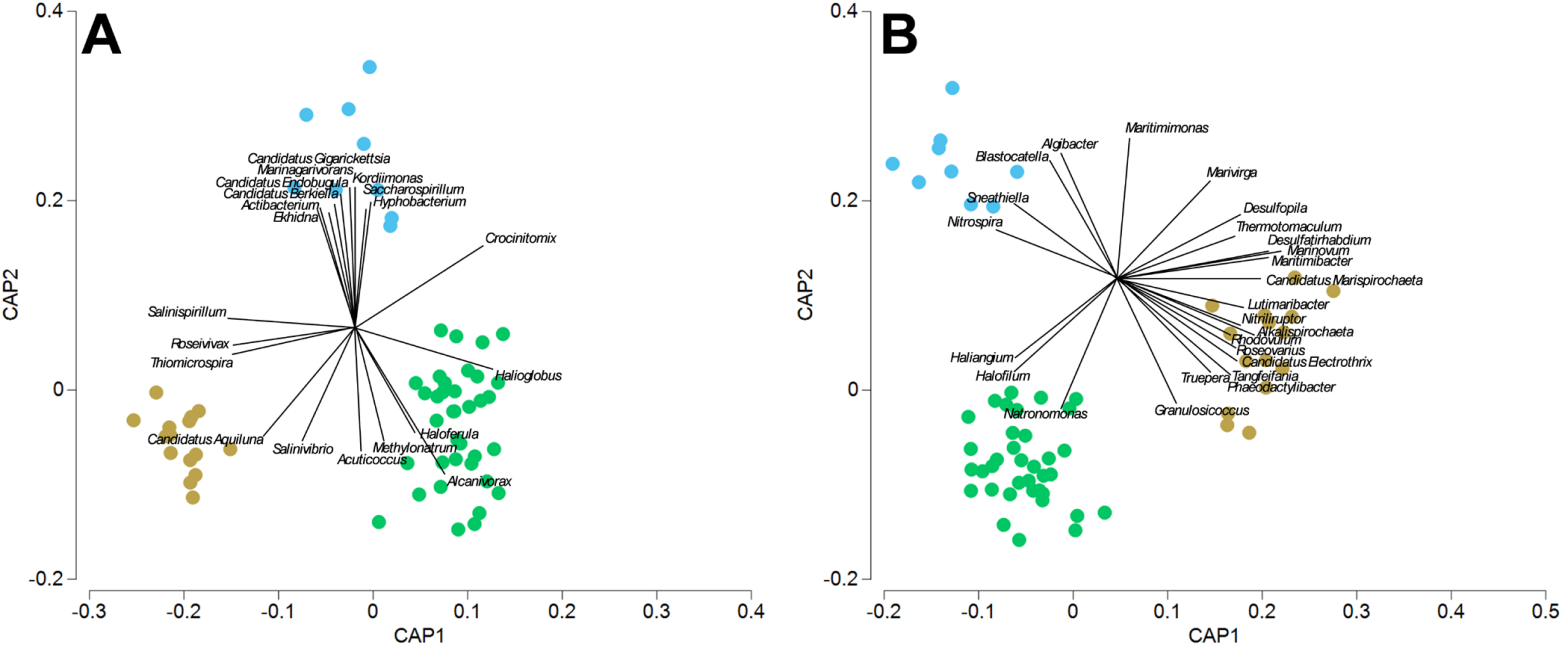
Canonical Analysis of Principal Coordinates (CAP) based on a Bray‐Curtis similarity matrix of the genus of Archaea and Bacteria in (A) water and (B) sediment samples at different stages of the life cycle of Ruppia that is, summer dormancy turions (blue), vegetative remnants (only roots present before vegetative regrowth; brown), and growing plants (leaves and roots present; green). Pearson correlation vectors (r > 0.5) represent the genera of Archaea and Bacteria (vectors) driving the differences between the environmental (water or sediment) microbial communities during these life stages of Ruppia. Samples were collected across four sampling trips at five sites (n = 3 per site per trip).

Canonical Analysis of Principal coordinates (CAP) analysis displayed clear spatial patterns revealing the influence of *Ruppia* leaves and roots on the sediment microbiota, with prokaryote communities distinctly organised according to the presence or absence of *Ruppia* leaves and/or roots (Figure 9B). A number of genera were positively or negatively correlated with the presence/absence of the *Ruppia* plant structures (Figure S4). The organisms that showed a positive correlation in the sediment when the leaves and roots of Ruppia plants were present are all known to be associated with high salinity environments typical of the seagrass habitats in this study. This is not surprising as the majority of the sampling trips were undertaken during the spring and summer months when the Coorong becomes hypersaline. A positive correlation was found between four genera in the sediment when *Ruppia* plants were not present. In the sediment when only roots were present 22 genera were found to be either positively or negatively correlated. Of all the genera, five belonged to the *Rhodobacteraceae* family which is prevalently identified in marine sediments due to their ability to oxidise sulfur, and/or ammonia or nitrite (Pujalte et al. 2014). Recently, *Rhodobacteraceae* has been identified as a key indicator of sediment ecosystem health and pollution (Rodríguez et al. 2021; Ramljak et al. 2024).

The water samples also reflected distinct groups in the community composition in the presence and/or absence of the *Ruppia* plant structures (Figure 9A). The microbial community structure explains the differences between the groups with 21 different genera positively or negatively correlated with the presence/absence of the *Ruppia* plants (Figure S5). Five water bacteria genera were positively correlated to the presence of both *Ruppia* leaves and roots. Eleven marine organisms were found to have either a positive or negative correlation when *Ruppia* plants were not present. When only the roots of the *Ruppia* plant were present, four bacterial genera were found to have a negative correlation and only one (*Roseivivax*) was identified as having a positive correlation. The presence of the genus *Roseivivax* (family *Rhodobacteraceae*) has been shown to significantly increase the settlement of coral larvae through the use of a chemical signal, although the signal has yet to be identified (Puglisi et al. 2019). In this study, *Roseivivax* levels rose in the months leading up to the vegetative regrowth life stage. Could this increase indicate that *Roseivivax* contributes to the regrowth of *Ruppia* under favourable conditions?

### 3.6 Algae

Filamentous aggregates attached to the seagrass plants (PAA) were only present in October 2020 in the Central and North Lagoons, while mats were observed at these sites in October and December 2020. Filamentous algae that form large areas of dense surface mats were observed to be almost exclusively associated with the presence of aquatic macrophyte habitat (Lewis et al. 2022). When investigating the correlation of filamentous algae (i.e., presence of PAA and mats, presence of mats, no filamentous algae) on the microbiota of the *Ruppia* community significant differences were observed (PERMANOVA *p* < 0.001; Figure 7D). A distinctive pattern is evident in the structure of the microbial communities when investigating the effect of the presence of PAA and mats of filamentous algae in the water (Figure 7D–F). The ordination according to the condition of filamentous algae is stronger for the water (Figure 7E; CAP 1 correlation *r* = 0.754) and sediment microbiota (Figure 7F; CAP 1 correlation *r* = 0.719) compared to the communities associated with Ruppia (Figure 7D; CAP 1 correlation *r* = 0.571). Several classes of Archaea and Bacteria were significantly correlated (*p* < 0.05) to the CAP axes and illustrated dissimilarities between the different filamentous algae conditions.

### 3.7 Microbial Community Structure Associated With Seagrass Health

The composition of the root‐associated microbiota is an important indicator of seagrass health and better understanding these seagrass root‐microbial interactions could allow us to optimise the seagrass microbiota and increase future restoration chances of success. Martin et al. (2020) have developed microbial indicators using 16S rRNA gene sequences of the seagrass root microbiota of *Halophila ovalis* in Western Australia, with the aim of obtaining early warning indicators of stress. Here, we assess the applicability of these indicators to assessing the health of the *Ruppia* community of the Coorong. This comparison was made possible as we used the same primers 341F–806R as Martin et al. (2020) that target the V4 hypervariable region of the 16S rRNA gene to amplify bacterial taxa.

Out of the 32 genera listed as indicators of ‘healthy’ seagrass root microbiota, 20 were present in our dataset, and of the 30 genera listed as indicators of ‘stressed’ seagrass root microbiota, 15 were present in our dataset (Table 1). To test the categorisation between ‘healthy’ and ‘stressed’ seagrass root microbiota, SIMPER and CAP analyses were conducted. These analyses compared the microbiota associated with the different conditions of *Ruppia* during our study: Leaves and roots are present (healthy), only roots are present, Turions are present. The CAP1 axis of the CAP analysis distinctively ordinates the communities according to the observed condition of *Ruppia*, as observed for the root microbiota (Figure 6A; CAP 1 correlation *δ*_1_^2^ = 0.716, *p* < 0.001) and for the sediment microbiota (Figure 6B; CAP 1 correlation *δ*_1_^2^ = 0.882, *p* < 0.001). Our dataset captures a substantial representation of key microbiota indicators associated with both healthy and stressed seagrass roots. This provides valuable insights into the microbial diversity of seagrass root ecosystems and lays a foundation for understanding how microbial communities influence seagrass health.

**TABLE 1:** List of the genera associated with ‘healthy’ and ‘stressed’ indicators of seagrass health according to Martin et al. (2020), and their presence in the root and sediment microbiome associated with the Ruppia community in the Coorong.

Genus	Indicators (Martin et al. 2020)	Sediment microbiome SIMPER	Root microbiome SIMPER
***Amphritea***	**X**		
**BD1‐7 clade**	**X**		
*Caldithrix*	X		
***Candidatus* Alysiosphaera**	**X**		
*Candidatus Aquiluna*			X
*Candidatus Electrothrix*	X	X	
*Candidatus Marispirochaeta*		X	
*Candidatus Megaira*	X		
***Candidatus* Nitrosopumilus**	**X**	**X**	
*Candidatus Photodesmus*			X
*Candidatus Tenderia*	X		X
*Candidatus Thiobios*		X	X
*Candidatus Thiodiazotropha*	X	X	X
***Cohaesibacter***	**X**		
*Deferrisoma*	X		
*Desulfobacca*		X	
*Desulfobacter*		X	
*Desulfobacterium*		X	
*Desulfobulbus*		X	
*Desulfoconvexum*			X
*Desulfomicrobium*	X		
*Desulfomonile*	X		
*Desulfonema*	X		
*Desulforhopalus*		X	X
***Desulfosalsimonas***		**X**	
*Desulfosarcina*		X	
***Desulfospira***	**X**		
*Desulfotignum*		X	
*Desulfovermiculus*		X	X
*Desulfovibrio*			X
*Geothermobacter*	X	X	
*Labilibacter*	X		X
*Methyloceanibacter*		X	X
***Methylophaga***	**X**		**X**
***Methylotenera***	**X**		
***Neorhizobium***	**X**		
***Ornatilinea***		
*Pseudoalteromonas*	X		X
***Pseudohongiella***	**X**		
***Reinekea***	**X**		**X**
SEEP‐SRB1	X		
SEEP‐SRB4		X	X
*Sulfitobacter*			X
*Sulfurimonas*		X	X
***Sulfurospirillum***	**X**		
*Sulfurovum*		X	
*Sva0081 sediment group*			
*Thalassotalea*	X		

*Note:* The genera listed here for the root and sediment microbiome associated with the Ruppia community were selected via SIMPER analysis and explained 20% of the dissimilarity between samples. In our study, we considered ‘healthy’ seagrass when both leaves and roots were present. Genera in orange and bold have only been associated with ‘healthy’ states of *H. ovalis* and here of the Ruppia community. Genera that are blue and underlined are only associated with the absence of roots and leaves in the Ruppia community.

### 3.8 Potential Functioning of the Seagrass, Sediment and Water Microbiota

**FIGURE 10:**
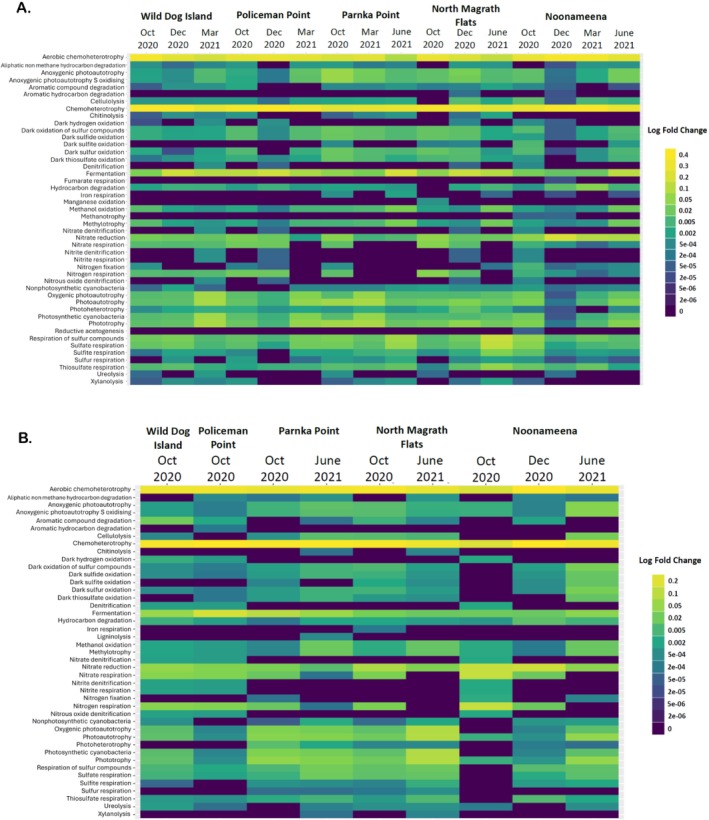
Heatmap depicting the metabolic and ecological functions of bacteria and archaea associated with *Ruppia* (A) roots and (B) leaves as inferred by FAPROTAX, based on the relative abundance of ASVs across the Coorong sampling sites during the different sampling periods. Differential abundance analysis was performed using DESeq2 (*n* = 3 per site per period).

Microorganisms are involved in crucial biogeochemical process within each environment, here we inferred the process using FAPROTAX (Louca et al. 2016). Fifty‐eight functional groups were represented and 6259 ASVs were assigned to at least one group (14.93% of ASVs). Of the putative biogeochemical cycle functions, 44 were identified in the water samples, 47 in the sediment samples, 46 in the root samples (Figure 10A), 43 on the leave samples (Figure 10B), and 52 in the filamentous algae samples. The most common functions across the water and sediment samples were chemoheterotrophy and aerobic chemoheterotrophy as well as autotrophic functions, phototrophy and photoautotrophy, whereas in the *Ruppia* samples (leaves and roots) chemoheterotrophy and aerobic chemoheterotrophy. Significant differences in putative functions were identified across sites within each sample type using DESeq2 (adjusted *p* < 0.05).

Microbial communities are vital for ecosystem health and have a vital role in biogeochemical processes, as well as in the food web. Microbial functions reflected seasonal differences in the biogeochemical state of the water that were evident across the Coorong lagoons (Figure S6A). During spring, two processes were significantly different (adjusted *p* < 0.05) across all the sites: Functions associated with the nitrogen cycle showed a decrease the further away from the inflow of fresh water from the Murray mouth, and an increase in photosynthesis was determined across the lagoons from south to north. In the summer, the functions attributed to photosynthesis increased across the lagoons: Noonameena (North lagoon) had the highest photosynthesis functions in comparison to the South lagoon and the Central lagoon, and functions attributed to the sulfur cycle decreased from the South lagoon (Wild Dog Island & Policeman Point) to the North lagoon (Noonameena; Figure S6A). During autumn, the functions associated with dark sulfur oxidation as part of the sulfur cycle decreased (Figure S6A). The sulfur cycle functions decreased with increasing geographical distance from the Murray mouth and the inflow of fresh water. In the winter, two processes were significantly different (adjusted *p* < 0.05) across all sites: the sulfur cycle functions belonging to the dark sulfur oxidation decreased in each lagoon with increasing geographical distance from Noonameena (North lagoon), and the functions attributed to photosynthesis increased across each lagoon with decreasing geographical distance to Noonameena (Figure S6A).

In the sediment samples, seasonality is evident across all the sites as described in the FAPROTAX functions. During spring, the functions associated with the sulfur cycle, especially the dark oxidation of sulfur compounds, were significantly different (adjusted *p* < 0.05) across all the lagoons (Figure S6B). In the summer, the functions of the sulfur cycle and fermentation were significantly different (adjusted *p* < 0.05) with the sites in the South lagoon being down regulated whereas sites in the Central and North lagoons were upregulated (Figure S6B). In the sediment, the sulfate reduction and fermentation work in balance within the sulfur cycle, as its balance is temperature dependent (Finke and Jørgensen 2008). During winter, the sulfur cycle functions appeared to be the metabolic process that was significantly different (adjusted *p* < 0.05) across all the lagoons (Figure S6B).

A number of biogeochemical functions were found to be significantly different (adjusted *p* < 0.05) in the filamentous algae samples between the Central and North lagoons during spring and summer (Figure S6E).

A rich and diverse microbiome found in the seagrass ecosystem has a key role in the biogeochemical cycles, especially the role that they have in assisting seagrass to cope with abiotic and biotic stress. In the leaf samples, of note is the oxidation of sulfur and related compound functions which during winter were higher in the central lagoon sites in comparison to the north lagoon (Noonameena).

In the root samples, during the summer the sulfur cycle, especially the dark oxidation of sulfur compounds was significantly different (adjusted *p* < 0.05) across the lagoons and the downregulation was evident in the South lagoon and Central lagoons while the cycle was upregulated in the North lagoon. During the autumn period, the nitrogen cycle functions showed changes across all the lagoons including nitrate reduction, nitrate respiration, nitrogen fixation, and nitrogen respiration. These functions were upregulated in the South and Central lagoons and downregulated in the North lagoon. In the winter, the sulfur cycle was also significantly different (adjusted *p* < 0.05) between the Central and North lagoons; however the functions upregulated were sulfate respiration, sulfite respiration, sulfur respiration and thiosulfate respiration.

The impact of eutrophication on seagrass ecosystems may be influenced by the composition and activity of sediment microbial communities (Wang et al. 2020). Mosley et al. (2023) reported that hypersaline conditions in the Coorong system have led to the loss of benthic ecosystems, contributing to sediment eutrophication. This process enhances nutrient availability through intensified nitrogen and sulfur cycling and organic matter mineralisation, which in turn increases seagrass exudation and leads to oxygen depletion (Liu, Jiang, et al. 2017). The resulting anoxic conditions promote the accumulation of hydrogen sulfide, creating a toxic environment for seagrass (Conte et al. 2021). Our findings suggest that the sediment microbiome plays a role in these processes, potentially exacerbating eutrophication‐related stress. This is supported by the presence of microbial taxa involved in nutrient cycling—particularly nitrogen and sulfur transformations—as well as anaerobic processes, highlighting the potential influence of sediment microbial dynamics on seagrass health under eutrophic conditions.

Martin et al. (2020) established that “stressed” seagrasses were dominated by sulfur‐cycling microorganisms. Oxidising sulfur and related compounds is an effective method to protect the seagrass against phytotoxic H_2_S intrusion (Tarquinio et al. 2019; Ruiz‐Reynés et al. 2023; Fraser et al. 2023). H_2_S is well recognised as being harmful and one of the main causes of seagrass deaths (Conte et al. 2021; Fraser et al. 2023). Hypersalinity has been determined to be one of the causes of H_2_S intrusion in seagrasses resulting in phytotoxicity (Johnson et al. 2020; Sandoval‐Gil et al. 2022). Due to the unique nature of the Coorong the salinity gradient increases the greater the distance from the Murray mouth. The South lagoon (Wild Dog Island and Policeman Point) and the Central lagoon (Parnka Point and North Magrath Flat) receive less freshwater flow in comparison to the North lagoon (Noonameena), thereby contributing to the higher salinity conditions (Mosley et al. 2023).

## 4 Conclusion and Perspectives

This study provides the first detailed insight into the microbial communities associated with the *Ruppia* community in the Coorong, highlighting the potential of microbial composition as a bioindicator for seagrass health. The strong correlation between microbial diversity and turbidity, as well as the presence of *Ruppia*, emphasises the ecological significance of these microbes in influencing plant health and ecosystem stability. Identifying key biofilm‐associated bacteria such as the genera belonging to *Rhodobacteraceae* offers a promising avenue for monitoring environmental changes that may stress the *Ruppia* community.

These findings open up new possibilities for enhancing seagrass restoration efforts by leveraging beneficial bacteria identified within the microbial communities. Work by Celdrán et al. [96] has demonstrated the efficacy of inoculating seagrass leaves with bacteria that would promote its growth. Targeting specific bacteria that is, *Phaeobacter* and *Roseivivax* to improve plant resilience, as well as prevent harmful algal blooms, could play a crucial role in the ecological restoration of the Coorong. Further research on the role of these bacteria, especially in mitigating environmental stressors, will be valuable for optimising restoration strategies and sustaining the health of seagrass ecosystems.

## Author Contributions

**Tamar Jamieson:** investigation, writing – original draft, methodology, writing – review and editing, formal analysis, data curation, validation, visualization, supervision. **Mohsen Chitsaz:** methodology, writing – review and editing, formal analysis, investigation, validation. **Angélique Gobet:** investigation, writing – review and editing, methodology, supervision, resources. **Michelle Waycott:** conceptualization, investigation, funding acquisition, writing – review and editing, project administration, supervision, validation, visualization. **Sophie C. Leterme:** conceptualization, investigation, funding acquisition, methodology, writing – review and editing, project administration, data curation, supervision, resources, visualization, validation.

## Funding

This work was supported by the South Australian Government's Healthy Coorong, Healthy Basin Program, 2019–2022 and by the ARC Training Centre for Biofilm Research and Innovation which is funded by the Australian Research Council (IC220100003).

## Conflicts of Interest

The authors declare no conflicts of interest.

## Supporting information


**Figure S1:** Pielou's evenness calculated for the communities of archae and bacteria present in each sample type (i.e., sediment, Ruppia roots, water, Ruppia leaves, plant associated mats, floating filamentous algae mats, and plant associated aggregates [PAA]) at each sampling site. Error bars represent the standard deviation to the mean.
**Figure S2:** Physico‐chemical parameters monitored at the five sampling sites in October 2020 (A), December 2020 (B), March 2021 (C), and June 2021 (D). Sampling site 1, Wild Dog Islands; site 2, Policeman Point; site 3, Parnka Point; site 4, North Magrath Flats; site 5, Noonameena. (*n* = 3 per site per trip).
**Figure S3:** Classes of bacteria identified in SIMPER analysis that explain most of the dissimilarities between trips and the sample types. Sampling site: 1, Wild Dog Islands; site 2, Policeman Point; 3, Parnka Point; site 4, North Magrath Flats; site 5, Noonameena. *n* = 3 per site per trip.
**Figure S4:** Bacteria genera in the sediment samples that were identified (Pearson correlation *r* > −0.5) as driving the differences between the life stages of Ruppia at the life stages of the Ruppia that is, no leaves and no roots, no leaves only roots present, and leaves and roots present. Samples were collected across four sampling trips at five sites (*n* = 3 per site per trip).
**Figure S5:** Bacteria genera in the water samples that were identified (Pearson correlation *r* > −0.5) as driving the differences between the life stages of Ruppia at the life stages of the Ruppia that is, no leaves and no roots, no leaves only roots present, and leaves and roots present. Samples were collected across four sampling trips at five sites (*n* = 3 per site per trip).
**Figure S6:** Heatmap representation of the log fold changes of the functional groups which showed significant difference (*p* < 0.005) between the five collection sites for (A) the water samples, (B) sediment samples, (C) *Ruppia* roots, (D) *Ruppia* leaves, and (E) filamentous algae samples. Shades of colour represent the log fold change increase and decrease (see colour scale). *n* = 3 per site per trip.

## Data Availability

The data that support the findings of this study are available on request from the corresponding author. The data are not publicly available due to privacy or ethical restrictions.
