# Supplementary material for: Seasonal Fluctuations of the Seagrass Holobiont under Contrasting Environmental Conditions
Source: Environ Microbiol Rep. 2026 Jan 23;18(1):e70239. doi: 10.1111/1758-2229.70239 (PMC12831020; doi:10.1111/1758-2229.70239)
Supplement: Supplementary file 1 — Figure S1: Pielou's evenness calculated for the communities of archae and bacteria present in each sample type (i.e., sediment, Ruppia roots, water, Ruppia leaves, plant associated mats, floating filamentous algae mats, and plant associated aggregates [PAA]) at each sampling site. Error bars represent the standard deviation to the mean. Figure S2: Physico‐chemical parameters monitored at the five sampling sites in October 2020 (A), December 2020 (B), March 2021 (C), and June 2021 (D). Sampling site 1, Wild Dog Islands; site 2, Policeman Point; site 3, Parnka Point; site 4, North Magrath Flats; site 5, Noonameena. (n = 3 per site per trip). Figure S3: Classes of bacteria identified in SIMPER analysis that explain most of the dissimilarities between trips and the sample types. Sampling site: 1, Wild Dog Islands; site 2, Policeman Point; 3, Parnka Point; site 4, North Magrath Flats; site 5, Noonameena. n = 3 per site per trip. Figure S4: Bacteria genera in the sediment samples that were identified (Pearson correlation r > −0.5) as driving the differences between the life stages of Ruppia at the life stages of the Ruppia that is, no leaves and no roots, no leaves only roots present, and leaves and roots present. Samples were collected across four sampling trips at five sites (n = 3 per site per trip). Figure S5: Bacteria genera in the water samples that were identified (Pearson correlation r > −0.5) as driving the differences between the life stages of Ruppia at the life stages of the Ruppia that is, no leaves and no roots, no leaves only roots present, and leaves and roots present. Samples were collected across four sampling trips at five sites (n = 3 per site per trip). Figure S6: Heatmap representation of the log fold changes of the functional groups which showed significant difference (p < 0.005) between the five collection sites for (A) the water samples, (B) sediment samples, (C) Ruppia roots, (D) Ruppia leaves, and (E) filamentous algae samples. Shades of colour [file EMI4-18-e70239-s001.docx]

**Supplementary Material**

Seasonal fluctuations of the seagrass holobiont in an environment with contrasting environmental conditions.

Tamar Jamieson^a,b*^, Mohsen Chitsaz^a^, Angélique Gobet^c^, Michelle Waycott^d,e^, Sophie C. Leterme^a,b*^

^a^ College of Science and Engineering, Flinders University, Bedford Park, South Australia

^b^ ARC Training Centre for Biofilm Research and Innovation, Flinders University, Bedford Park, SA 5042, Australia

^c^ MARBEC, Univ Montpellier, CNRS, Ifremer, IRD, Sète, France

^d^ School of Biological Sciences, Faculty of Science, The University of Adelaide, Adelaide, South Australia

^e^ State Herbarium of South Australia, Botanic Gardens and State Herbarium, Hackney Road, Adelaide, South Australia

^*^ T. Jamieson and S.C. Leterme have contributed equally to this manuscript

Corresponding authors: T. Jamieson [tamar.jamieson@flinders.edu.au](mailto:tamar.jamieson@flinders.edu.au) and S.C. Leterme [sophie.leterme@flinders.edu.au](mailto:sophie.leterme@flinders.edu.au)

Supplementary methods

*Water Quality*

Water sampling was undertaken approximately 10-15 cm under the surface of the water column. Measurements of temperature (°C) and salinity (PSU) were recorded in triplicates using a multi- parameter probe (Hanna, HI 98195). Dissolved Oxygen (DO) was recorded in triplicates using a Handy Polaris (Oxyguard, H01P). Water samples (500 ml) for Turbidity (NTU) were collected in triplicates and analysed immediately upon collection using a waterproof Turbidimeter (Thermo Scientific EUTech TN-100).

Samples for dissolved nutrient analysis, i.e., ammonium, nitrite, nitrate, and phosphate, were collected from the subsurface waters in triplicates and filtered on 0.45 μm syringe filters (Sarstedt, Germany) prior to storage at -20°C until analysis. Analyses of all dissolved nutrient concentrations were measured simultaneously and carried out following published methods (Leterme et al., 2015), using a SKALAR SFA nutrient analyser.

*Amplicon Formation*

For each DNA extract, the universal primer pair 515F/806R was used to amplify the prokaryotic V4 region of the 16S rRNA gene which has a median amplicon length of 292 base pairs (bp), with a range of 290–295 bp. The primer pairs also contained Nextera adapter sequences (specific to the sequencing platform) as well as individual barcode sequences for identification of each sample. PCR reactions were performed for each sample, and each reaction included about 1 ng of template DNA, 0.32 μM of each primer, 2 U Q5 Hot Start High-Fidelity DNA Polymerase (New England Biolabs), 1× Q5 reaction buffer (New England Biolabs), 0.8 mM dNTP (Combined; Promega) and MilliQ water for a total volume of 50 μl. The conditions for PCR were as follows: 98°C for 1 min to denature the DNA, with 30 cycles at 98°C for 30 seconds, 55°C for 30 seconds, and 72°C for 30 seconds, and a final extension of 10 min at 72°C to ensure complete amplification.

Successful DNA amplification was confirmed using gel electrophoresis and the PCR products were sent to the Australian Genome Research Facility (AGRF, Melbourne, Australia) for sequencing.

*Sequencing and bioinformatics pipeline*

DNA sequence data was generated using Illumina MiSeq™ System (2 x 250 bp paired-end sequencing) at the Australian Genome Research Facility (AGRF, Melbourne, Australia). DADA2 package (version 1.22; Callahan et al. 2016) in R studio v.4.6.1 (R v.4.1.3; https://www.r-project.org/) was used for sequence analysis and produced amplicon sequence variants (ASVs). Raw sequences were demultiplexed with barcodes and adaptors removed by AGRF. The paired-end 16S rRNA gene raw sequencing data comprised 228 samples which generated 27,104,165 total reads. The cutadapt program (Martin, 2011) was used to remove the primers from the amplicon reads. Sequences were filtered and trimmed following standard filter parameters, with the quality score of the sequence of less than or equal to 2 and with the maximum expected errors for forward reads equalling 2, and 5 for reverse reads. All sequences which matched the phiX genome were discarded. After filtering and trimming, the 228 samples comprised of 24,955,834 sequences. The paired-end sequences were merged followed by denovo chimera removal. Taxonomy was inferred (98% similarity) for the 16S rRNA gene sequences using the SILVA database release 138.1 (https://www.arb-silva.de/documentation/release-1381/; Quast et al. 2013). Singletons and taxon such as Eukaryota, Mitochondria and Chloroplast were removed from the dataset.

*Statistical Analysis*

To assess species diversity, Pielou’s evenness, and species richness, community diversity index were computed using the PRIMER v.7 software +PERMANOVA add on (Clarke and Gorley, 2006; Anderson et al. 2008). Data collected were not normally distributed after applying a Shapiro-Wilk test. To test for significant differences between means of parameters, nonparametric, Kruskal-Wallis tests (Zar, 1996) were calculated using the IBM SPSS 22.0 Software.

Multivariate statistics were performed using PRIMER v.7 software +PERMANOVA add on. Environmental data were examined in draftsman scatter plots to ascertain whether some variables were highly correlated to each other, and if the assumptions made about the data were valid. Draftsman plots were examined before and after log(x+1) transformation and normalisation. A Principal Component Analysis (PCA) was used to explore the water quality parameters contributing to the differentiation between the sampling sites. This ensured the data were approximately multivariate-normally distributed before performing a PERMutational ANalysis Of VAriance (PERMANOVA) using a Euclidean distance resemblance matrix to test for significant differences in overall water quality using a 2-factor design (month – fixed and location – fixed).

The microbial community data were transformed using log(x+1) to meet assumptions of normal distribution and homoscedasticity. Dissimilarity was assessed between pairs of groups (e.g., leaves versus roots, roots versus sediment, leaves versus water) using the SIMPER method (Anderson et al. 2008). A Bray-Curtis similarity matrix was calculated between the communities and the data were then analysed by Principal Coordinate Analysis (PCoA). The six classes responsible for most of the dissimilarities between pairs were used to illustrate differences on the PCoA plot. We then used PERMANOVA to identify whether communities were significantly different between sampling sites and months. A Bray-Curtis similarity-based resemblance matrix was calculated on the communities. Canonical Analysis of Principal coordinates (CAP) based on Bray-Curtis similarities was used to plot the discrimination between the five sites, based on community composition. The BEST procedure was used to identify which environmental variables associated with the differences observed in the community composition. The BVSTEP procedure within BEST searches for high rank Spearman correlations between a similarity matrix of community composition and matrices of normalised environmental variables (Clarke and Gorley, 2006).


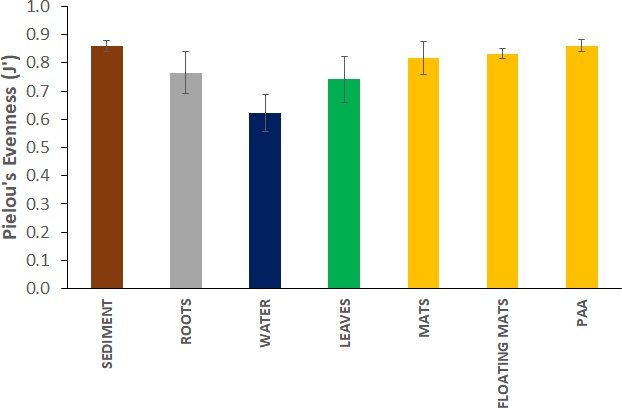


Figure S1. Pielou’s evenness calculated for the communities of archae and bacteria present in each sample type (i.e. sediment, Ruppia roots, water, Ruppia leaves, plant associated mats, floating filamentous algae mats, and plant associated aggregates (PAA)) at each sampling site. Error bars represent the standard deviation to the mean.


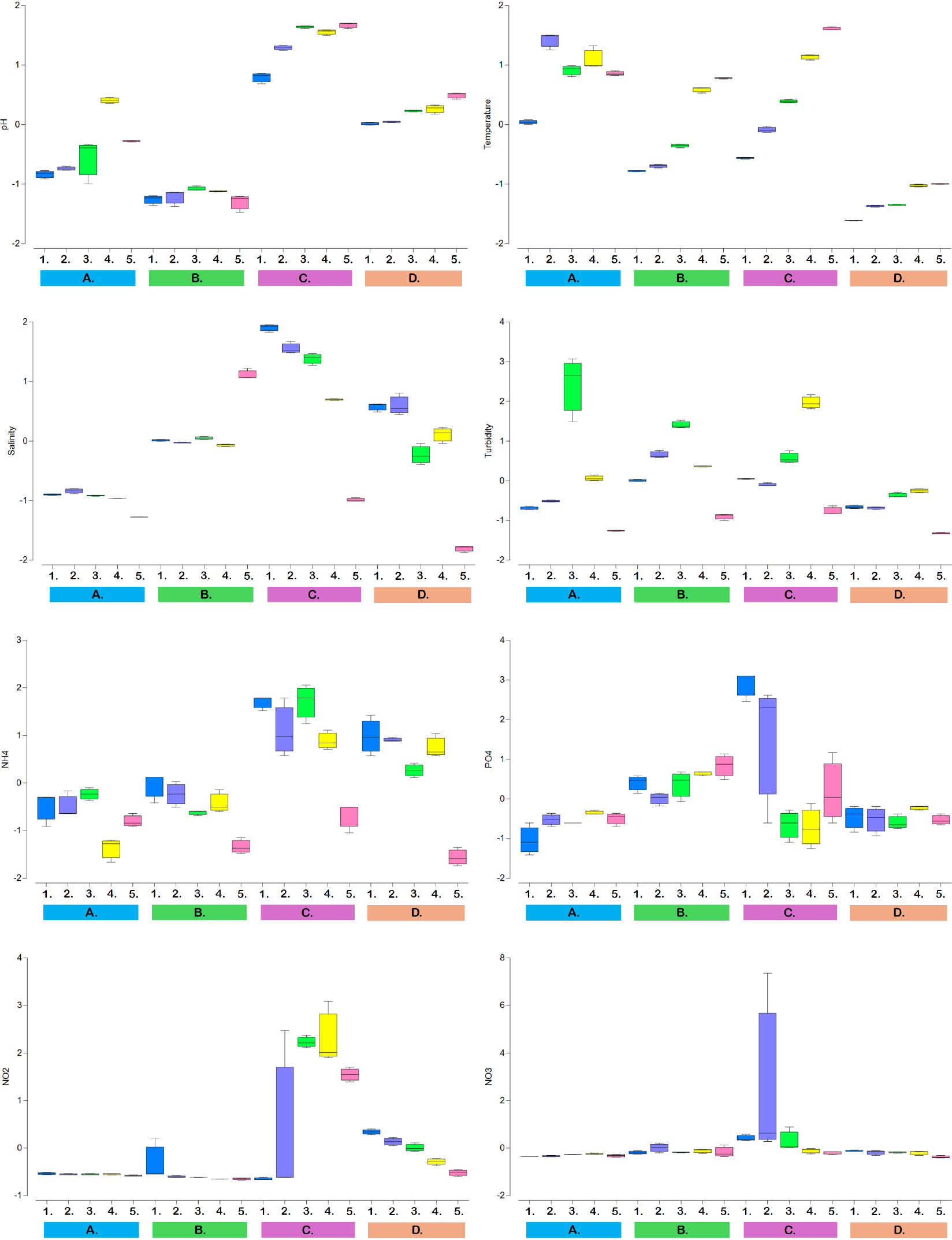


Figure S2: Physico-chemical parameters monitored at the five sampling sites in October 2020 (A), December 2020 (B), March 2021(C), and June 2021 (D). Sampling site 1, Wild Dog Islands; site 2, Policeman Point; site 3, Parnka Point; site 4, North Magrath Flats; site 5, Noonameena. (n = 3 per site per trip).


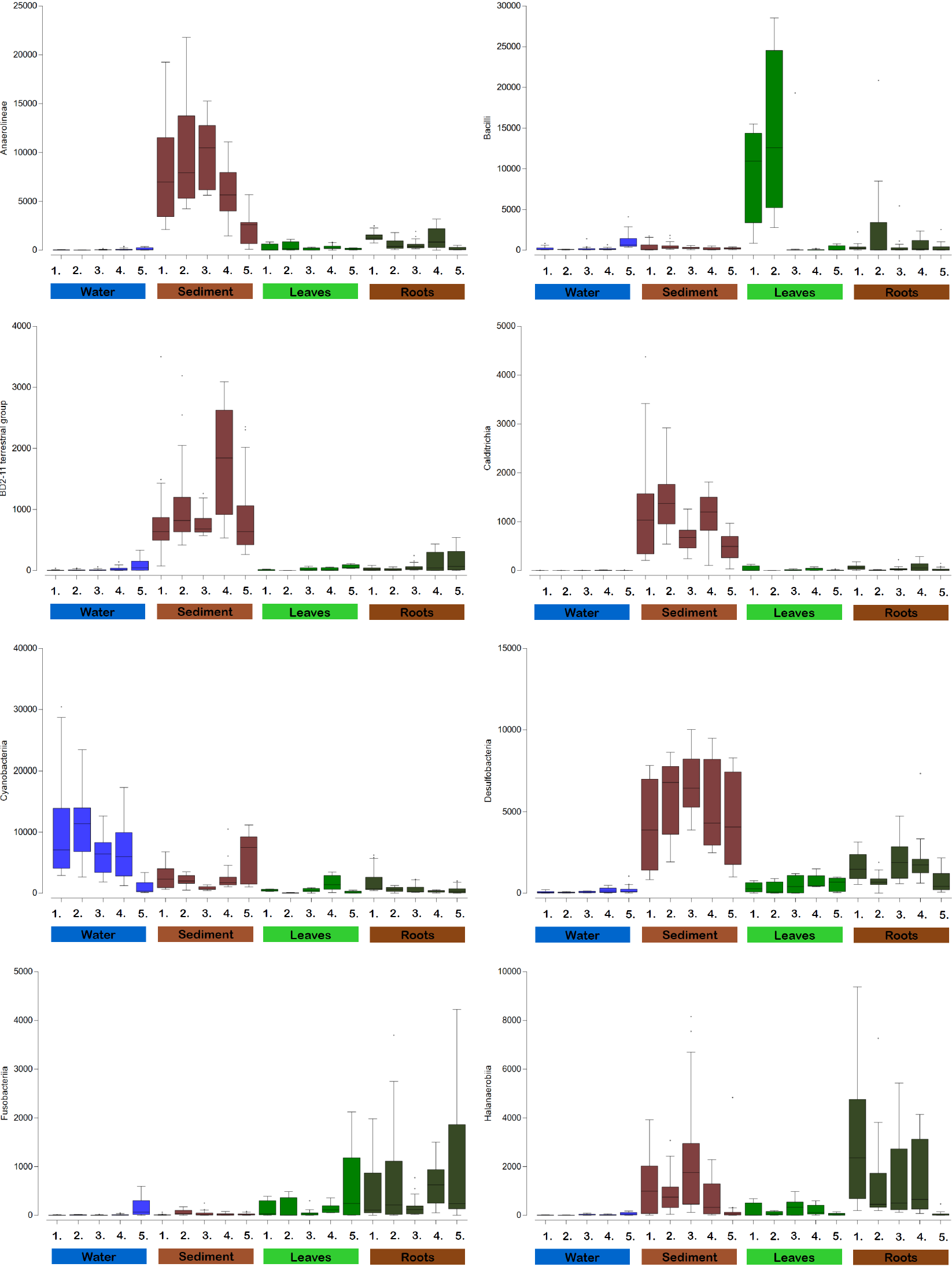


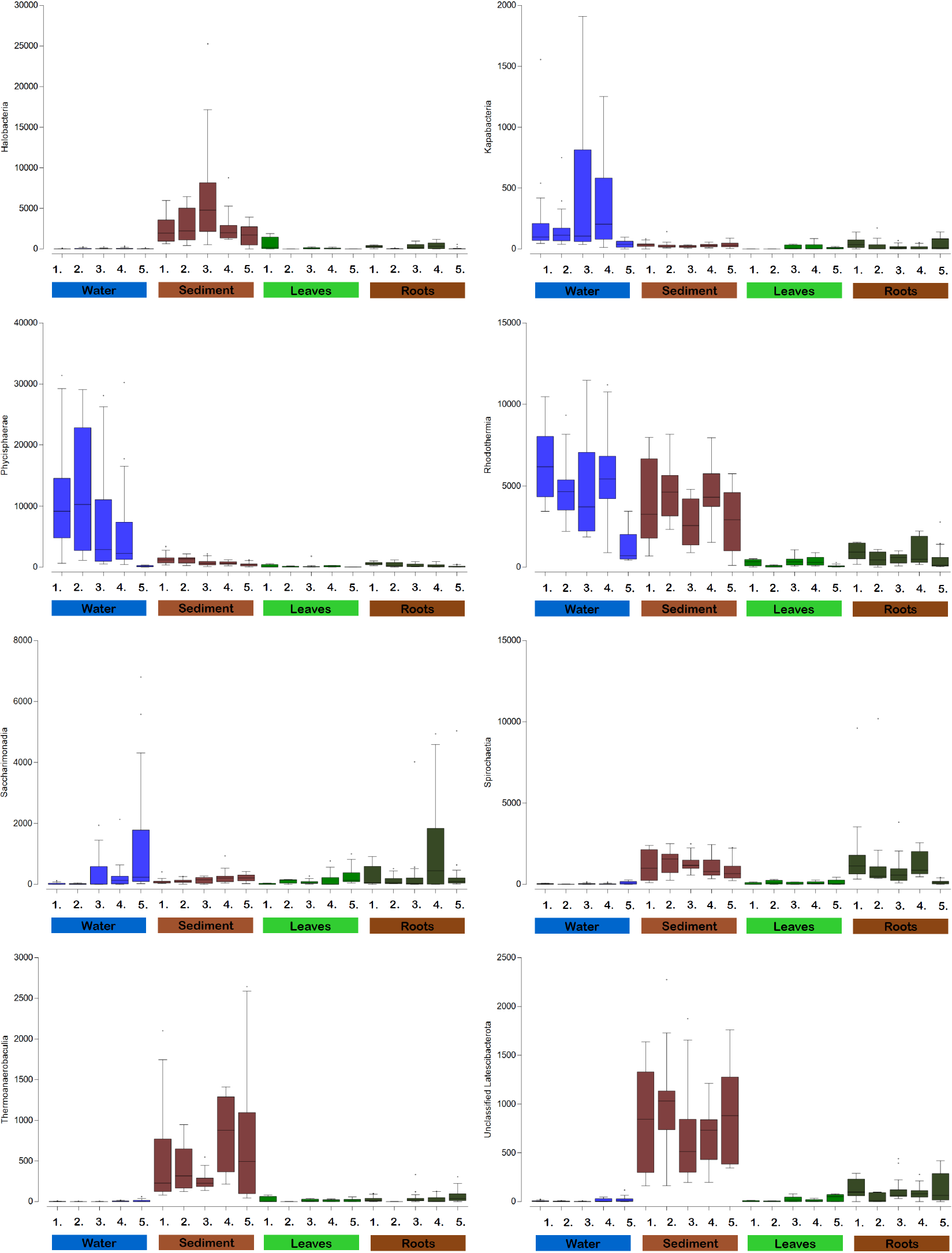


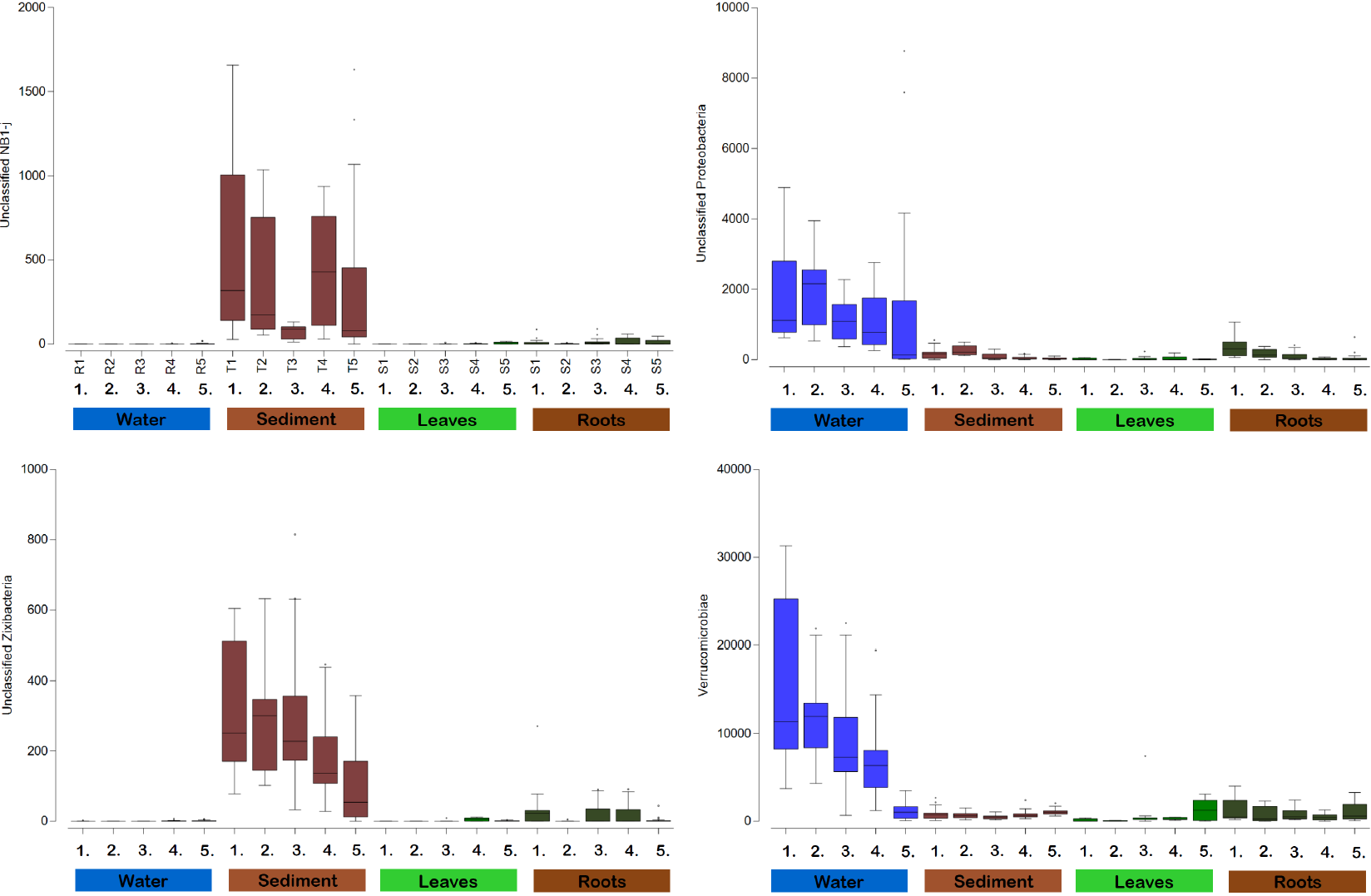


Figure S3: Classes of bacteria identified in SIMPER analysis that explain most of the dissimilarities between trips and the sample types. Sampling site: 1, Wild Dog Islands; site 2, Policeman Point; 3, Parnka Point; site 4, North Magrath Flats; site 5, Noonameena. n = 3 per site per trip.


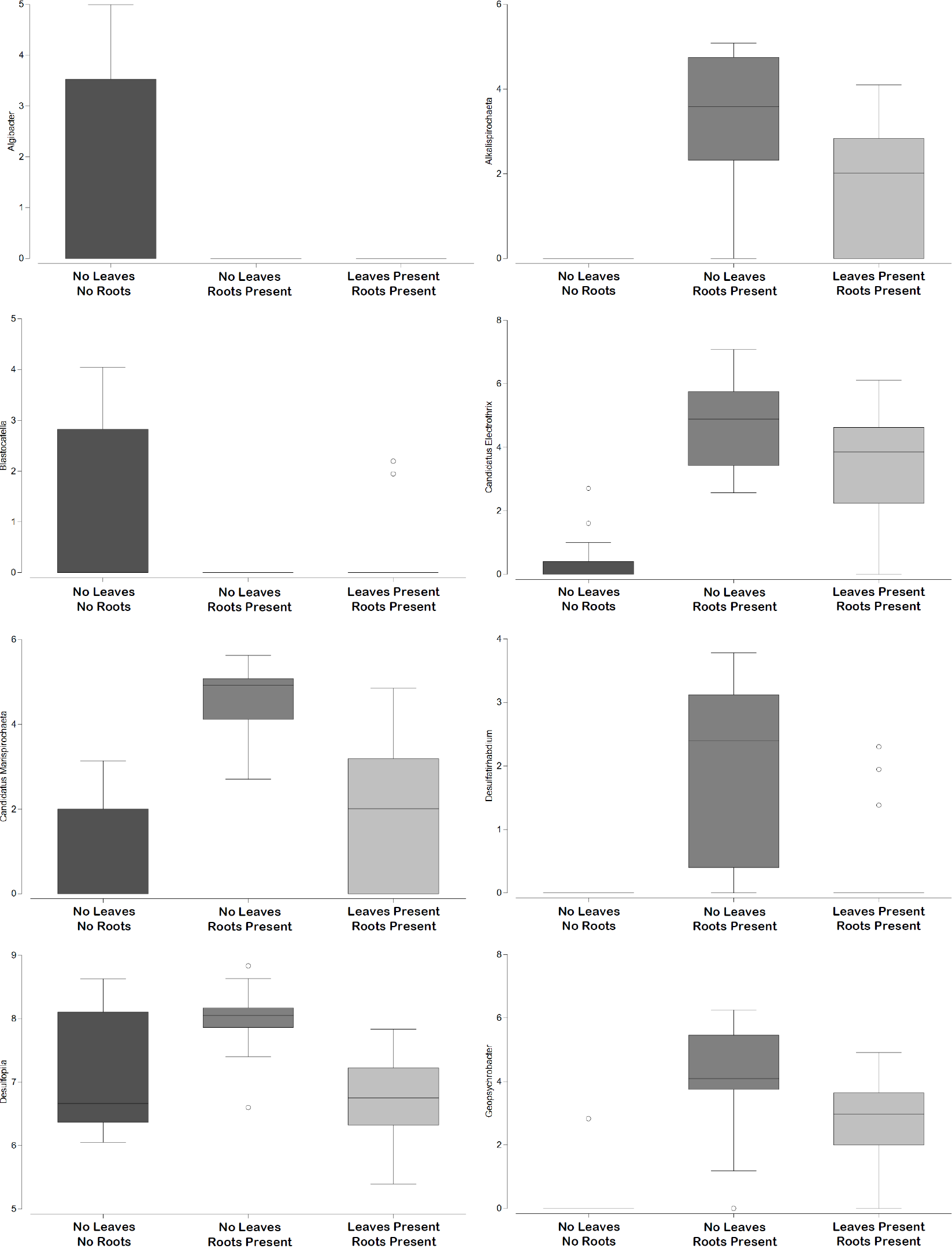


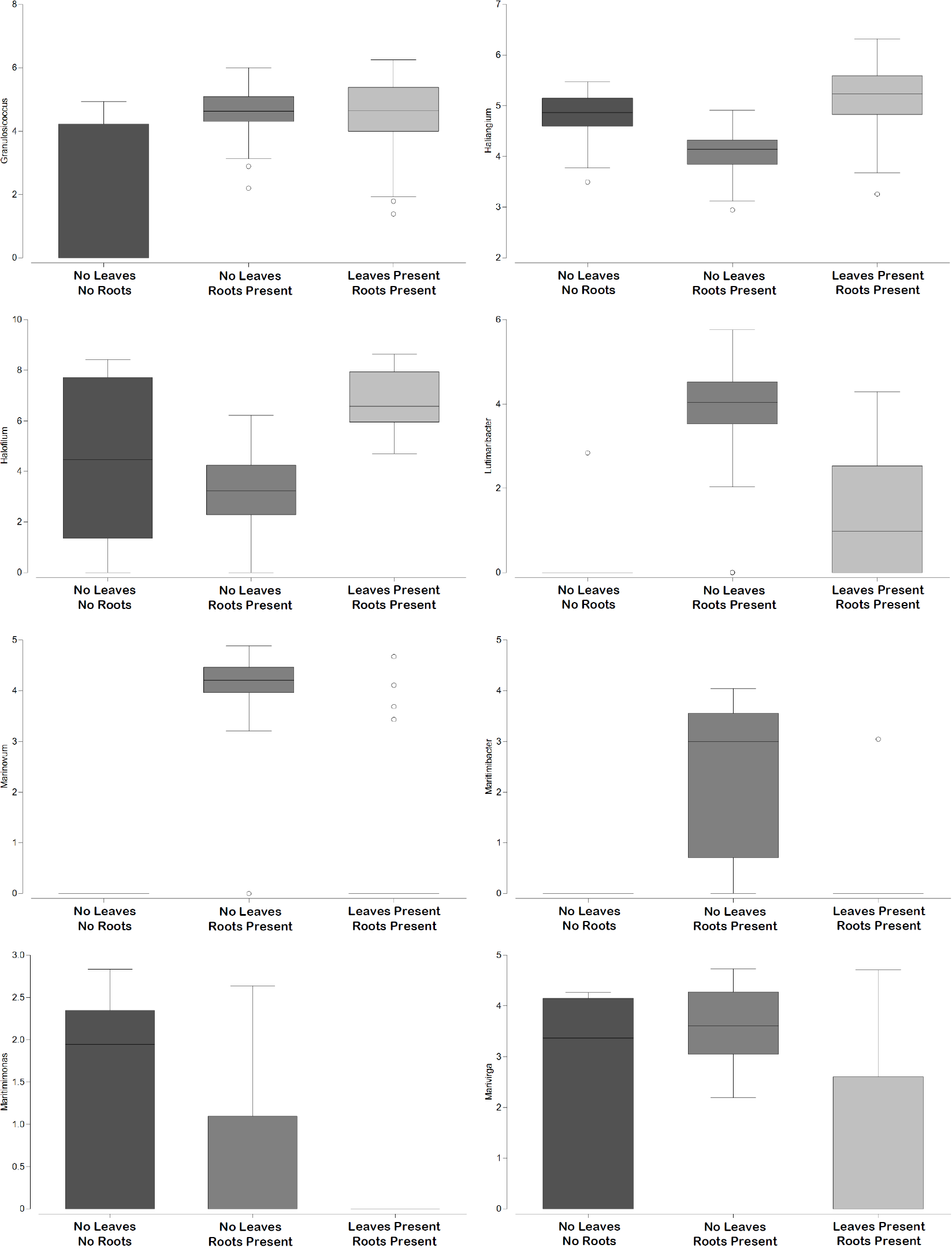


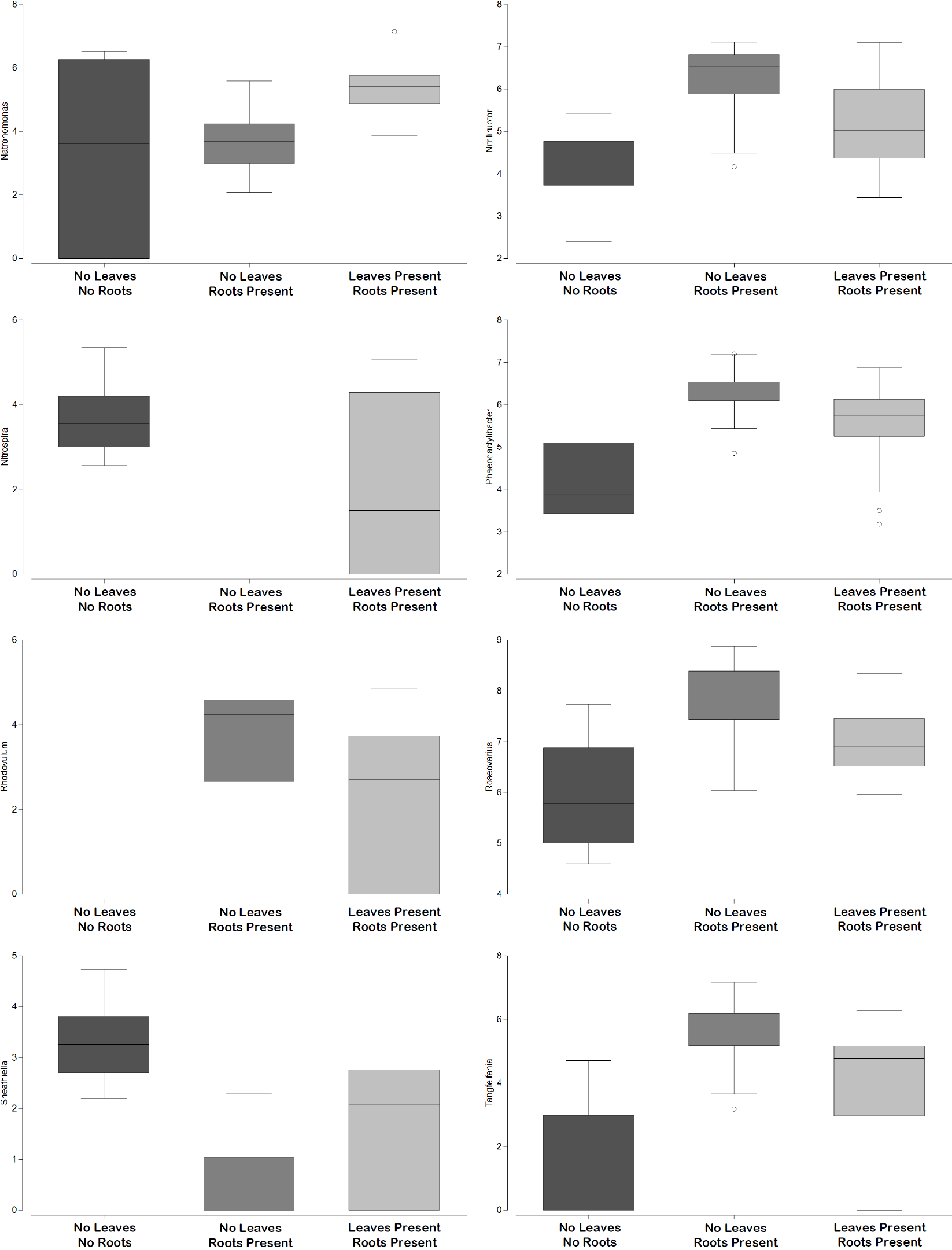


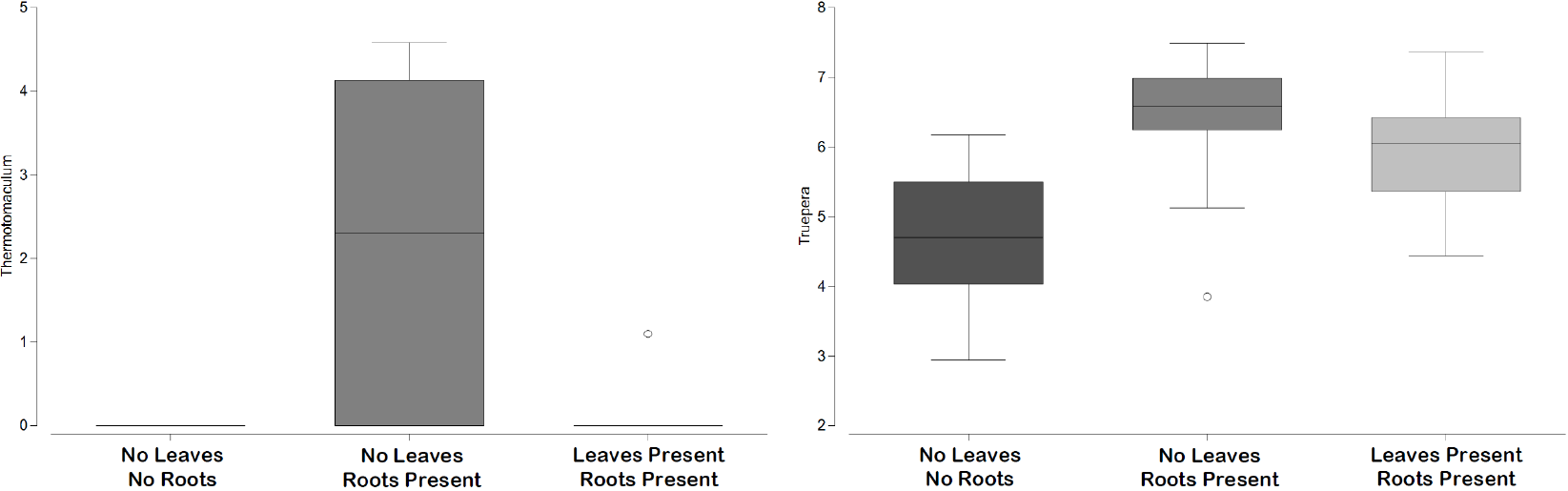


Figure S4: Bacteria genera in the sediment samples that were identified (Pearson correlation r>-.5) as driving the differences between the life stages of Ruppia at the life stages of the Ruppia i.e., no leaves and no roots, no leaves only roots present, and leaves and roots present. Samples were collected across four sampling trips at five sites (n = 3 per site per trip).


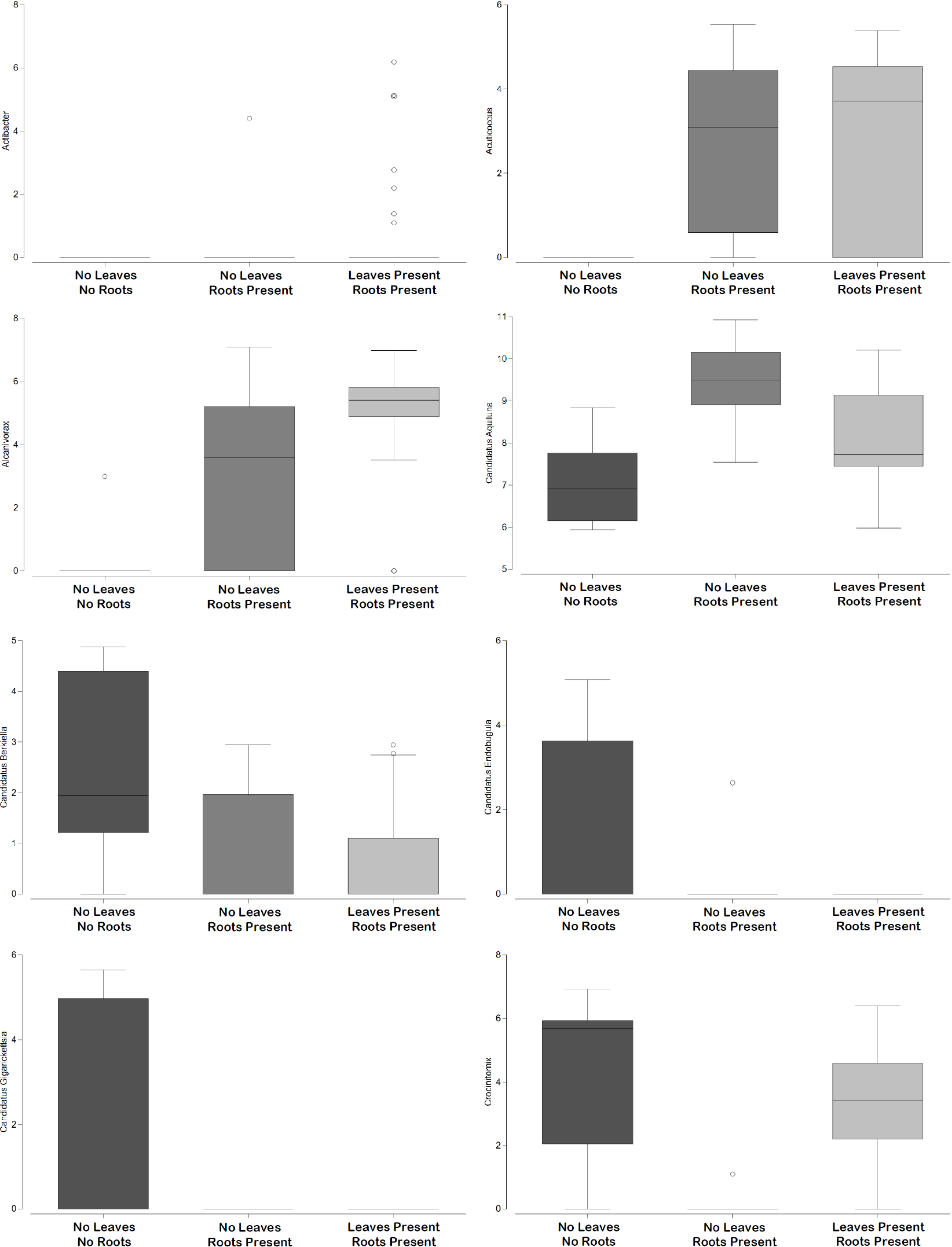


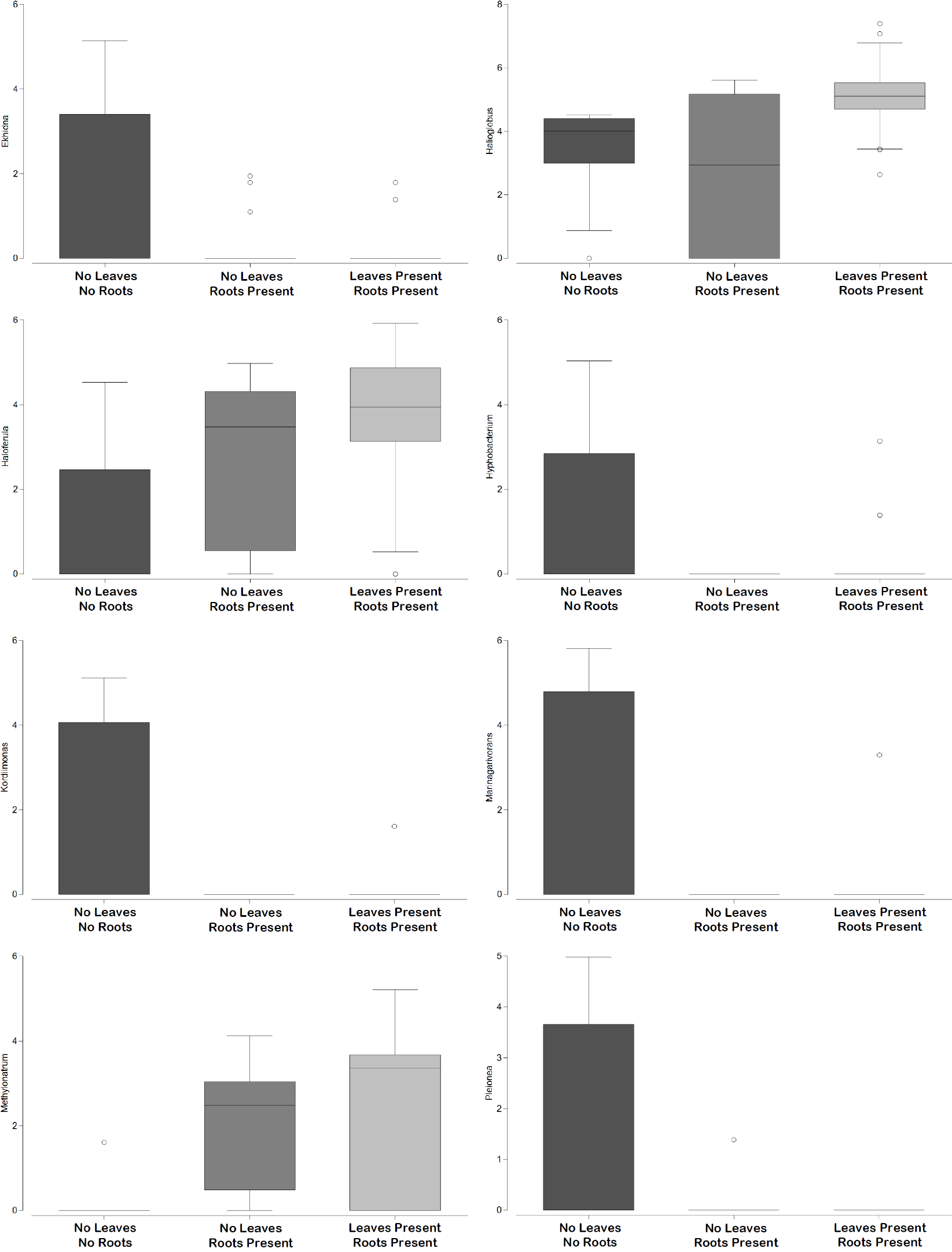


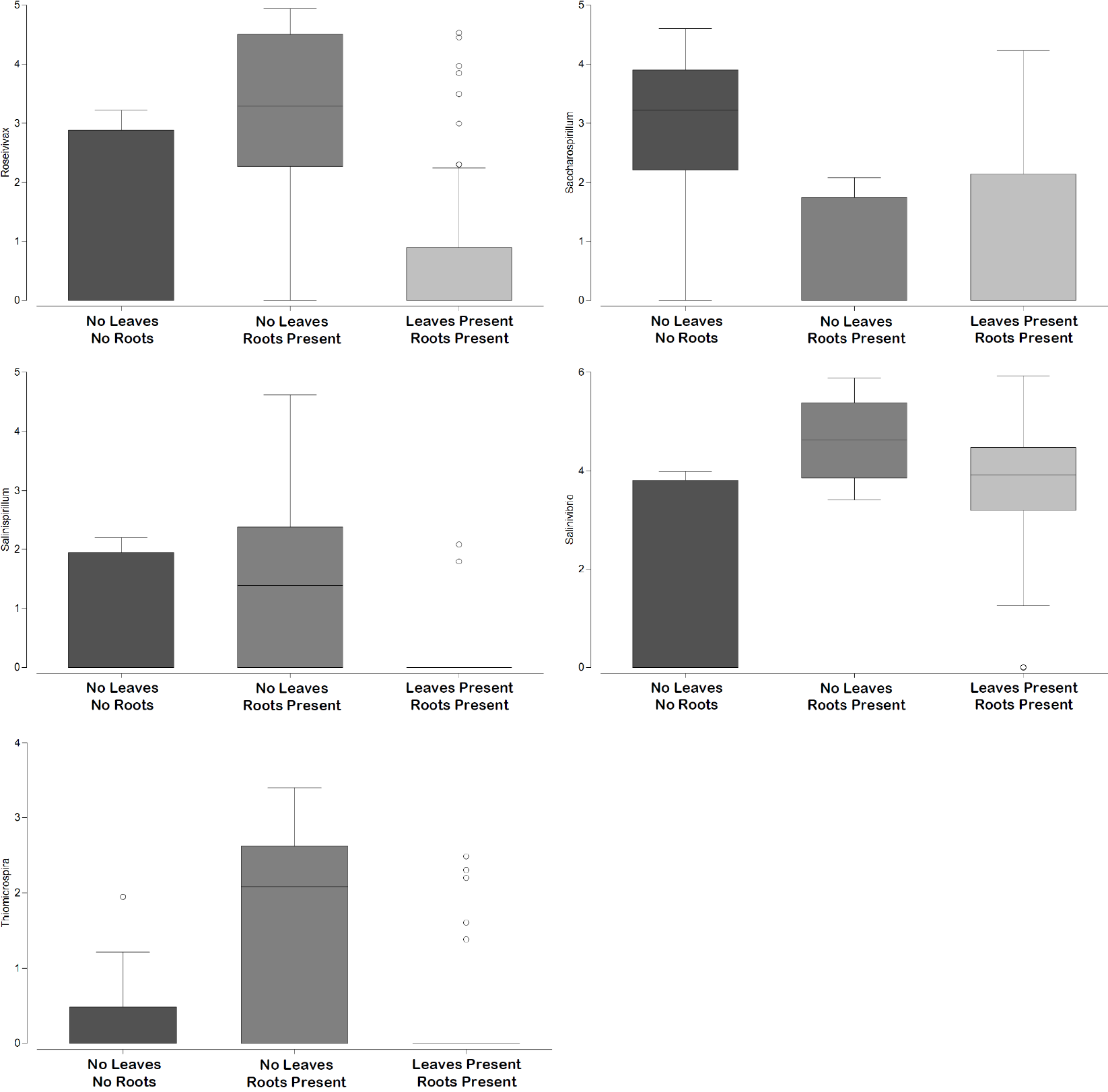


Figure S5: Bacteria genera in the water samples that were identified (Pearson correlation r>-.5) as driving the differences between the life stages of Ruppia at the life stages of the Ruppia i.e., no leaves and no roots, no leaves only roots present, and leaves and roots present. Samples were collected across four sampling trips at five sites (n = 3 per site per trip).


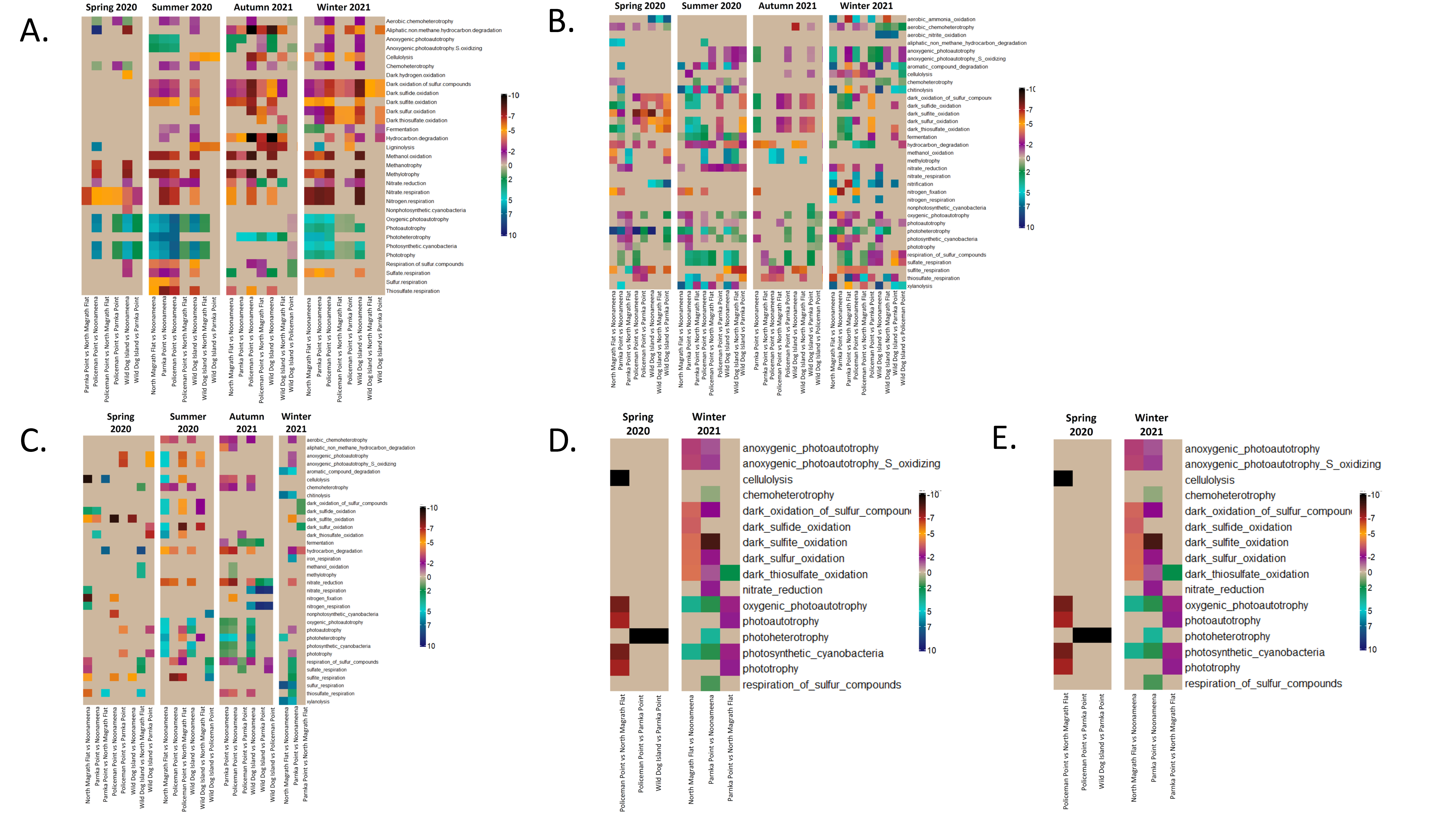


Figure S6: Heatmap representation of the log fold changes of the functional groups which showed significant difference (*p* < 0.005) between the five collection sites for (A) the water samples, (B) sediment samples, (C) *Ruppia* roots, (D) *Ruppia* leaves, and (E) filamentous algae samples. Shades of colour represent the log fold change increase and decrease (see colour scale). n = 3 per site per trip.

*References*

Anderson M, Gorley RN, and Clarke RK. Permanova+ for Primer: Guide to Software and Statistical Methods. Primer-E Limited. 2008

Callahan BJ, McMurdie PJ, Rosen MJ, Han AW, Johnson AJ, Holmes SP. DADA2: High-resolution sample inference from Illumina amplicon data. Nat Methods. 2016 Jul;13(7):581-3.

Clarke KR, and Gorley RN. Primer. PRIMER-e, Plymouth. 2006.

Leterme, S.C., Allais, L., Jendyk, J., Hemraj, D.A., Newton, K., Mitchell, J. and Shanafield, M., 2015. Drought conditions and recovery in the Coorong wetland, South Australia in 1997–2013. Estuarine, Coastal and Shelf Science, 163, pp.175-184.

Martin M. Cutadapt removes adapter sequences from high-throughput sequencing reads. EMBnet. journal. 2011 May 2;17(1):10-2.

Quast C, Pruesse E, Yilmaz P, Gerken J, Schweer T, Yarza P, Peplies J, Glöckner FO. The SILVA ribosomal RNA gene database project: improved data processing and web-based tools. Nucleic Acids Res. 2012 Nov 27;41(D1):D590-6.

Zar JH. Circular distributions: descriptive statistics. Biostatistical analysis. 1996:519-611.
